# A genomic analysis of mouse models of breast cancer reveals molecular features of mouse models and relationships to human breast cancer

**DOI:** 10.1186/bcr3672

**Published:** 2014-06-05

**Authors:** Daniel P Hollern, Eran R Andrechek

**Affiliations:** 1 Department of Physiology, Michigan State University, 2194 Biomedical and Physical Sciences Building, 567 Wilson Rd., East Lansing, MI 48864, USA

## Abstract

**Introduction:**

Genomic variability limits the efficacy of breast cancer therapy. To simplify the study of the molecular complexity of breast cancer, researchers have used mouse mammary tumor models. However, the degree to which mouse models model human breast cancer and are reflective of the human heterogeneity has yet to be demonstrated with gene expression studies on a large scale.

**Methods:**

To this end, we have built a database consisting of 1,172 mouse mammary tumor samples from 26 different major oncogenic mouse mammary tumor models.

**Results:**

In this dataset we identified heterogeneity within mouse models and noted a surprising amount of interrelatedness between models, despite differences in the tumor initiating oncogene. Making comparisons between models, we identified differentially expressed genes with alteration correlating with initiating events in each model. Using annotation tools, we identified transcription factors with a high likelihood of activity within these models. Gene signatures predicted activation of major cell signaling pathways in each model, predictions that correlated with previous genetic studies. Finally, we noted relationships between mouse models and human breast cancer at both the level of gene expression and predicted signal pathway activity. Importantly, we identified individual mouse models that recapitulate human breast cancer heterogeneity at the level of gene expression.

**Conclusions:**

This work underscores the importance of fully characterizing mouse tumor biology at molecular, histological and genomic levels before a valid comparison to human breast cancer may be drawn and provides an important bioinformatic resource.

## Introduction

Breast cancer is a heterogeneous disease with significant mortality associated with metastatic progression. Classification subdivides human breast cancer into six categories including Luminal A, Luminal B, HER2+, Basal, Claudin-low and normal-like [1]. Recent work suggests additional subclasses exist within each intrinsic subtype including three basal subtypes with striking differences in overall survival [2]. Further, The Cancer Genome Atlas (TCGA) and the Encyclopedia of DNA Elements (ENCODE) projects show remarkable variability in genetic alterations beyond gene expression both across and within subtypes of human breast cancer. Together these genomic analyses demonstrate the complex nature of human breast cancer.

To more readily study mechanisms leading to breast cancer, research has turned to the mouse as a model. Mouse models of breast cancer have employed various methods of initiation, including mouse mammary tumor virus (MMTV) infection, chemical mutagenesis and genetically engineered mice (GEM). This pioneering work identified and tested the role of many oncogenes in breast cancer. With the insertion of MMTV into the genome, numerous key oncogenes were uncovered [3,4]. The later development of MMTV driven transgenics allowed for development of spontaneous models. With the identification of human epithelial growth factor receptor 2 (HER2) amplification in human breast cancer [5,6], the observation that MMTV driven expression of the activated rat form of HER2 (NeuNT) resulted in breast cancer reinforced the importance of HER2 as a driving oncogene [7]. More recently, models have been refined to include tissue specific activation resulting in gene amplification, analogous to human HER2+ breast cancer [8], as well as temporal control where transgene expression can be activated or inactivated [9].

Individual mouse models have been used to model aspects of human breast cancer and the selection of the appropriate model to compare to human breast cancer has been directed by phenotype or known genetic events. For instance, the MMTV-PyMT model is widely used to examine metastasis [10] while P53 knockout mammary epithelium transplanted into wild type hosts results in tumors with various genetic mutations [11]. Another aspect is the histological subtype associated with various tumors in GEM models and the metastatic ability can be altered with background [12]. Indeed, similarities between mouse models such as Neu and Wnt as well as their human counterparts have been previously noted [13,14]. Importantly, in both human breast cancer and in many GEM models, there is significant histological heterogeneity [15-17]. These attributes illustrate the importance and utility of mouse models to examine breast cancer.

With the number and variety of GEM models, it is important to consider how accurately these various systems model human breast cancer. Initial studies using intrinsic clustering revealed similarities between mouse models and human breast cancer, albeit in a limited number of samples [18]. Yet, a more detailed characterization of a larger number of p53 null tumors revealed a variety of subtypes with strong similarities to human breast cancer [11], revealing the importance of examining a large number of samples to capture tumor heterogeneity and variability. Further, expanding the number of Myc induced tumors revealed that a subpopulation of Myc induced tumors had similarities to claudin-low human breast cancer [19]. Taken together, recent comparative studies [11,17,19-22] highlighted a clear need for a comprehensive examination of the genomic features of mouse models of breast cancer and their relation to human breast cancer. To this end, we assembled an expansive dataset of mouse models of breast cancer. This dataset reveals the genomic heterogeneity of mouse models and offers a predictive resource for essential cell signaling pathways. Importantly, all comparisons between all models are made available with our report. These data demonstrate the similarities and differences of the various subtypes of mouse models to the key subtypes of human breast cancer and underscore the necessity for an informed choice of the appropriate mouse model for studying specific types of human breast cancer.

## Methods

### Combination of datasets

Datasets (GSE10450, GSE11259, GSE13221, GSE13231, GSE13259, GSE13553, GSE13916, GSE14226, GSE14457, GSE14753, GSE15119, GSE15263, GSE15632, GSE15904, GSE16110, GSE17916, GSE18996, GSE20465, GSE20614, GSE21444, GSE22150, GSE22406, GSE23938, GSE24594, GSE25488, GSE27101, GSE30805, GSE30866, GSE3165, GSE31942, GSE32152, GSE34146, GSE34479, GSE6453, GSE6581, GSE6772, GSE7595, GSE8516, GSE8828, GSE8863, GSE9343, GSE9355 GSE37954, GSE2034, GSE2603, GSE4922, GSE6532, AND GSE14020) were downloaded from Gene Expression Omnibus. E-TABM-683 and E-TABM-684 were downloaded from Array Express. For Affymetrix data, Bayesian Factor Regression Methods (BFRM) [23] were used to combine datasets and remove batch effects [24]. Agilent data was merged with Affymetrix data using Chip Comparer [25] and Filemerger [26]. To remove platform effects between Affymetrix and Agilent data and batch effects between individual Agilent studies we used COMBAT [27,28]. Batch effects and batch correction were visualized by principle component analysis in Matlab (for code see Additional file 1).

### Data analysis

Unsupervised hierarchical clustering was done using Cluster 3.0 and exported using Java Tree View. The color scheme for the heatmap and sample legends were made using Matlab. Human breast cancer sample intrinsic subtypes were classified according to protocol [1]. Prior to clustering mouse models with human breast cancer, we clustered the human breast tumor samples on their own, to identify genes that would organize the breast tumors according to their intrinsic subtype in the combined dataset. We used these genes to filter the mouse and human combined gene expression dataset for unsupervised hierarchical clustering.

Significance analysis of microarrays [29] was used for fold change analysis. Settings for each comparison can be found in the excel download for each model (Additional files 2, 3). Gene ontology and TRANSFAC predictions were made using GATHER [30]. Gene set enrichment analysis was conducted using Genepattern [31]. The gene-set describing mammary cell-types was derived from [32].

Pathway activation was predicted according to previous studies [2,33]. For mouse samples, specific conditions for each pathway signature can be found in Additional file 4. For human breast tumor samples, pathway activation was predicted using Score Signatures [34] and conditions can be found [2]. Mixture modeling was implemented according to [2].

## Results

### Database assembly

We assembled a database containing 1,172 samples from mouse mammary tumor models, cell types and normal mammary gland. The major mouse models and descriptions are listed in Table 1. Within a number of these models, variants exist with different alleles, promoters, and genetic backgrounds. In assembling the database, we measured the non-biological variance between gene expression studies and batch correction with principle components analysis (PCA) (Additional file 5A-D). PCA demonstrated that normalization successfully removed artificial variance between datasets (Additional file 5B,D). As a control, we confirmed batch correction utilizing Neu-initiated tumors spanning the Affymetrix and Agilent platforms from several studies. Prior to normalization (Additional file 5E) PCA demonstrated that Neu tumors varied by platform. After correction, Neu tumors clustered together in PCA, demonstrating that artifactual variance has been removed (Additional file 5F). With platform and batch effects eliminated, we began to explore relationships in the mouse model database.

**Table 1:** List of mouse models in the dataset

**Model**	**Arrays**	**Promoter**	**Description**	**References**
Myc	319	MMTV WAP/Dox	Myc mammary tumors of various histological types, expression levels and stability with variable Kras mutations.	[15,19,20,35-38]
Neu	124	MMTV	Induction of adenocarcinomas with pulmonary metastasis.	[11,15,38-44]
PyMT	119	MMTV K6/RCAS MMTV/RCAS	Rapid induction of luminal-type mammary tumors with pulmonary metastasis.	[38,45-48]
SV40 Large T Antigen	107	C3 WAP	Induction of mammary tumors with similarities to human basal type breast cancer.	[11,38,49-51]
p53	92	Null	Tumors with similarities to human basal type breast cancer.	[11,38,52,53]
CreEtv6/NTRK3	63	WAP	Fusion oncoprotein transforms through activation of AP1.	[54]
MET	52	MMTV	Diverse histologies with similarities to human basal breast cancer.	[16]
BRCA/p53	46	WAP MMTV BLG	CKO of BRCA1 in a p53 null background. Tumors similar to human basal breast cancer.	[38,55]
Wnt	35	MMTV	Induction of mammary tumors with diverse gene expression patterns.	[38,56-58]
IGF-IR	26	MTB	Basal-like mammary tumors. Recurrent tumors resemble human claudin-low.	[59]
LPA	16	MMTV	ER positive, metastatic tumors.	[60]
Stat5	16	BLG	Induction of mammary tumors	NA
Brg1 (+/−)	14	Mutant	Heterogeneous breast cancers.	[61]
DMBA	12	Chemical	Mammary carcinomas with three phenotypes: adenocarcinoma, squamous cell carcinoma, and myoepithelial cell carcinoma.	[62]
Ras	10	MMTV	Induction of mammary tumors with rapid tumor onset.	[38]
Int3	9	WAP	Metastatic tumors.	[63]
RB/p107	7	CKO	Adeno and adenosquamous carcinomas similar to luminal B or basal.	[64]
APC CKO	6	K14-Cre	CKO results in adenocarcinomas with histological and molecular heterogeneity.	[65]
Autotaxin (ATX)	5	MMTV	ER + metastatic mammary tumors.	[60]
BRCA	5	CKO	Tumors similar to human basal type breast cancer.	NA
STAT1	5	Knockout	ERa + PR+, hormone dependent like human ERa + luminal.	[66]
Notch	4	Dox	Induction of mammary adenocarcinomas.	NA
PDK1	2	MMTV	Induction of mammary tumors	[67]
Normal Mammary Gland	47	Not Applicable	Normal mammary gland samples from FVB, BalbC, and CD1 genetic backgrounds.	[16,44,60]

CKO, conditional knockout; Dox, doxacycline inducible MMTV-Rtta system; MMTV, mouse mammary tumor virus.

### Gene expression heterogeneity in mouse models

**Figure 1:**
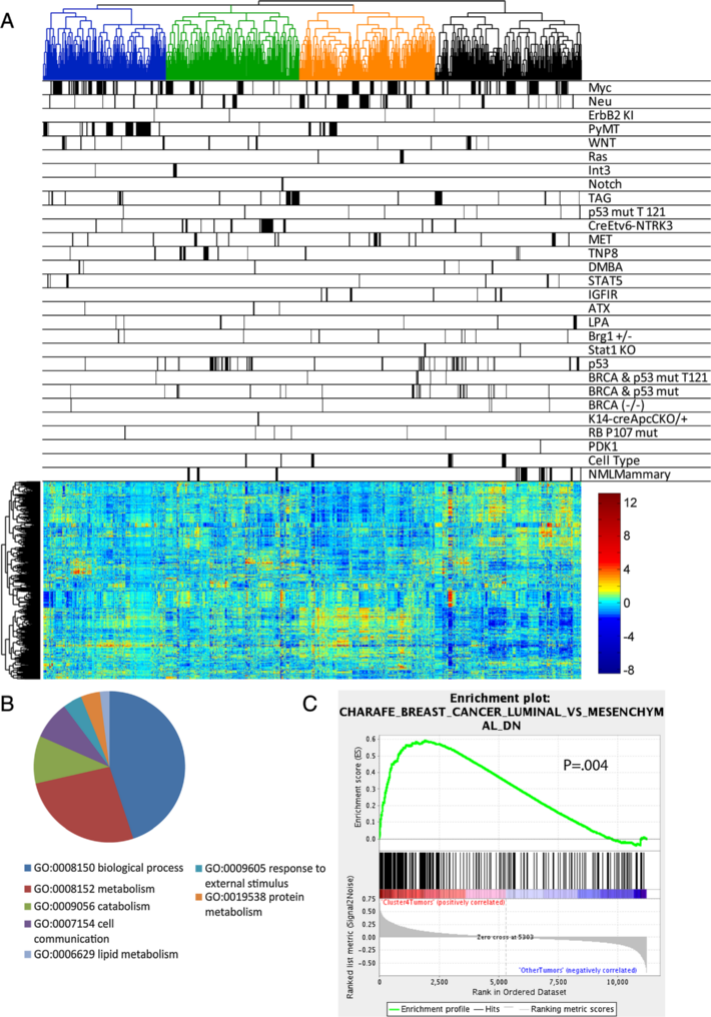
**Analysis of relationships between mouse mammary tumor models. (A)** The unsupervised hierarchical clustering analysis of gene expression data for mouse mammary tumors, cell types and normal mammary gland is shown. The dendrogram across the top illustrates relationships between samples and is color-coded to itemize the four main clusters. Below the dendrogram, black bars label samples from each corresponding model on the same line. Gene expression values are illustrated with the heatmap, according to the scale shown. The vertical dendrogram beside the heatmap illustrates genes with similar patterns of expression across the samples in the dataset. **(B)** The pie chart illustrates the gene ontologies of the genes that are significantly (q = 0, fdr = 0) over-expressed as identified by SAM in the blue cluster of tumors compared to tumors in other clusters. **(C)** The gene set enrichment plot comparing tumors from cluster 4 (black) to tumors in the other clusters shows significant enrichment for high expression of a gene set that defines mesenchymal breast cancer (*P* = .004). SAM, significance analysis of microarrays.

Using unsupervised hierarchical clustering, we examined mouse mammary tumors initiated by various oncogenes. Unsupervised hierarchical clustering generated four major clusters (Figure 1A). We observed remarkable variability in gene expression profiles, including within model heterogeneity. For example, Myc initiated tumors span each of the major clusters in the dendrogram. In contrast, some models show uniformity in gene expression from tumor to tumor, including Ras initiated tumors that ordered into a single cluster. Interestingly, there was significant interrelatedness between tumor models initiated with different oncogenes. Annotations for individual tumors revealed that similarities in tumor histology correlated with relationships in gene expression profiles. For example, MMTV-Myc, MMTV-Met and a subset of 7,12-dimethylbenz[a]anthracene (DMBA) induced tumors of the adenosquamous histology shared gene expression profiles. These data reveal mouse models with various levels of heterogeneity and illustrate some of the tumor phenotypes that drive relationships between different mouse models.

To define the characteristics of each cluster, we used Significance Analysis of Microarrays (SAM) to identify differentially regulated genes that define tumors within each cluster (Additional file 6). We interrogated gene lists for gene ontologies (Additional file 6). For instance, Figure 1B shows the gene ontologies for the upregulated genes in the blue cluster in Figure 1A. Ontological categories included genes involved in biological processes and metabolism. To refine these results, tumors from each cluster were examined with Gene Set Enrichment Analysis (GSEA) (Additional file 7). Focusing on tumors in the black cluster, GSEA showed enrichment for gene sets separating mesenchymal cells from luminal cells (Figure 1C, Additional file 8A), including low expression of Zeb1 target genes (Additional file 8B). Gene lists that define mammary stem cells demonstrated that this cluster also had a gene expression profile enriched for mammary stem cell-like features (Additional file 8C,D). In agreement, the majority of epithelial to mesenchymal transition (EMT) like tumors were observed in the black cluster (Figure 1A, Additional file 9). GSEA also demonstrated that tumors from the other clusters had gene expression profiles consistent with luminal cells (Additional file 10). For example, tumors within the blue cluster correlated with gene signatures for luminal progenitor cells and the orange cluster had similarities in gene expression to mature luminal cells. Together, these results define the characteristics of the tumors contained in the major clusters.

### Fold change analysis

**Figure 2:**
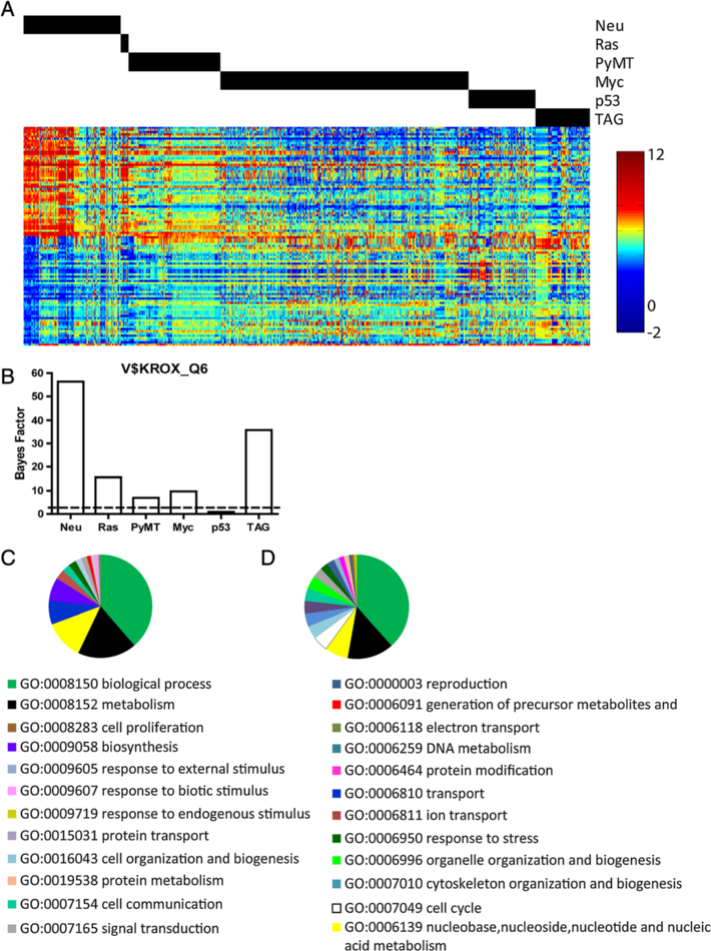
**Fold change analysis of Neu induced tumors compared to other tumor models. (A) **The expression pattern for the top 50 significantly (q = 0, fdr = 0) upregulated and down regulated genes for Neu-induced tumors as identified by SAM are illustrated with the heatmap. Above the heatmap, black bars denote the model each sample corresponds to. Expression levels are depicted according to the colorbar beside the heatmap. **(B)** The bar graph shows the bayes factor measuring the enrichment of predicted binding sites for the Krox family of transcription factors within upregulated genes from each model. The dotted line indicates a bayes factor of 2.0. **(C)** Gene ontologies for upregulated genes in Neu induced tumors are depicted in the pie chart according to the color-coded categories. **(D)** Gene ontologies for upregulated genes in TAG induced tumors are depicted in the pie chart according to the listed color-coded categories. SAM, significance analysis of microarrays; TAG, large T antigen.

Given that unique initiating events in the tumor models should cause characteristic responses associated with the tumor initiating event, we used SAM to identify genes significantly altered within each model compared to all other models (Additional file 2). Fold change differences were also calculated between the tumors within a model and normal mammary glands in the corresponding genetic background (Additional file 3). As an example, we determined fold change gene expression differences for Neu initiated tumors (Figure 2A). Collectively, SAM analysis provided a collection of genes that are differentially expressed in each model.

To identify possible transcription factors that could be active in mediating these gene expression changes, we annotated fold change results for each model using TRANSFAC (Additional file 2, 3). For example, for genes regulated by Neu (Figure 2A), we predicted that a significant number of genes had predicted binding sites for the Krox family of transcription factors (Figure 2B). The complete results for the transcription factor binding predictions are included in the additional data for each of the models.

**Figure 3:**
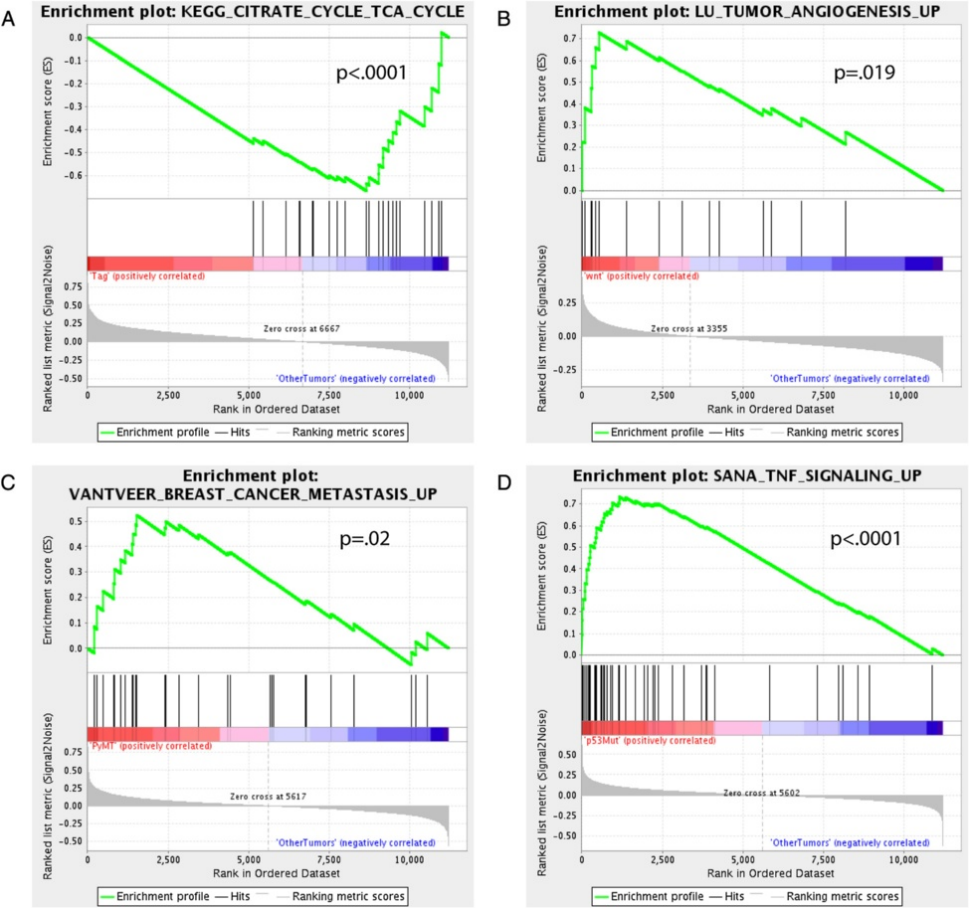
**Gene set enrichment analysis of mouse mammary tumor models. (A)** Gene set for genes involved in the TCA cycle are significantly enriched (*P* < .0001) for low expression in TAG tumors. **(B)** A gene set for genes upregulated during tumor angiogenesis are significantly enriched (*P* = .019) for high expression in Wnt induced tumors. **(C)** A gene set for genes upregulated in breast cancer metastasis is significantly enriched (*P* = .02) for high expression in PyMT induced tumors. **(D)** A gene set for genes that upregulated as a result of TNF signaling is significantly enriched (*P* < .0001) for high expression in p53 mutant tumors. PyMT, polyoma middle T; TAG, large T antigen; TCA, the citric acid cycle.

We also annotated fold change differences between each model using gene ontologies (Additional files 2, 3). As an example of the utility of the method, we examined the similarities and differences in gene ontologies in the Neu and TAG models (Figure 2C). Both Neu and TAG tumors featured biological processes, metabolism and nucleic acid-related metabolism as major ontological categories. Key differences included Neu tumors with genes related to transport, ion transport and biosynthesis, categories not found with TAG gene expression changes. TAG tumors had major ontologies representing genes involved in cell cycle, cell organization, cytoskeleton organization and biogenesis, and cell organization and biogenesis. To expand upon gene ontology results we compared each model to all other models and separately to normal mammary gland using GSEA (Additional file 11). This analysis predicted unique features for all models including specific information on metabolism, microenvironment, metastasis and possible pathway activation (Figure 3). For example, TAG tumors had down regulation of genes significantly enriched for the citric acid cycle TCA) (Figure 3A). Wnt tumors were predicted to have upregulation of tumor angiogenesis (Figure 3B). Not surprisingly, polyoma middle T (PyMT) tumors show enrichment for gene sets that predict metastasis (Figure 3C). Finally, GSEA results predicted that p53 mutant tumors may have increased TNF signaling activity (Figure 3D). Together, these results provide a catalogue of possible important features corresponding to the transcriptional outcomes of an initiating oncogene event.

### Pathway analysis

**Figure 4:**
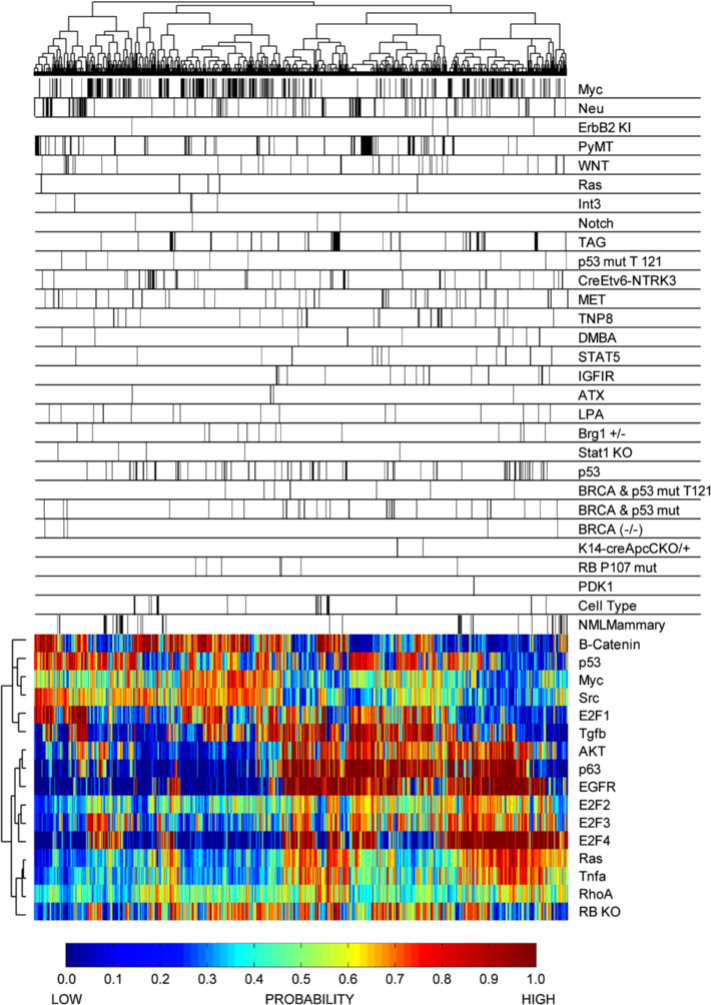
**Unsupervised hierarchical clustering of pathway activation predictions in mouse mammary tumors.** The dendrogram across the top illustrates the relationship between samples based on predicted pathway activation profiles. Below the dendrogram, the black bars mark tumor samples corresponding to the model listed on the same line. The heatmap illustrates the probability of pathway activation according to the color bar provided below the heatmap. The vertical dendrogram beside the heatmap illustrates pathways with similar predicted activity across the samples in the dataset.

To expand the predictive analysis, we utilized a gene signature approach to predict pathway activation across mouse mammary tumors. The pathway prediction relationships between the various models were organized with unsupervised hierarchical clustering (Figure 4). Using this approach, we noted a large degree of heterogeneity within models. Myc tumors showed extensive variation in pathway activation profiles, spanning the spectrum of clusters. To understand better the heterogeneity and pathway activity within each model, we viewed the pathway predictions on a model-by-model basis (Additional file 12). For example, in PyMT induced tumors, there is a significant difference in predicted pathway activity between tumors from a FVB and AKXD genetic background (Additional file 13). Myc induced tumors with an EMT or squamous histology had distinct predicted pathway activities relative to tumors with a papillary or microacinar histology (Additional file 14). In Neu-induced tumors, we observed a major difference in predicted pathway activity between Neu tumors using the MMTV promoter and a Tet-on system to drive oncogene expression (Additional file 15). Taken together, these data demonstrate that tumor type, genetic background, and promoter result in key differences in pathway activity.

To validate and illustrate the utility of pathway activation predictions for developing hypotheses about pathways that function in tumor progression, we identified models with clear pathway activity predictions. Previous genetic studies that correlate with these predictions are noted (Table 2). Demonstrating the validity of the gene signatures, we observe a large degree of agreement between pathways with predicted activity and results from previous investigations.

**Table 2:** Validation of pathway predictions

**Model**	**Pathway**	**Effect**	**References**
APC cKO	B-Catenin	Demonstrated high activation of β-catenin signaling in these tumors.	[65]
APC cKO	Myc	High levels of Myc demonstrated by IHC in these mammary tumors.	[65]
BRCA & P53 mut	EGFR	Using IHC, EGFR was shown to be overexpressed in this mouse model.	[68]
DMBA	Ras	Observation of H-Ras mutations in mammary hyperplastic outgrowths after treatment with DMBA.	[69]
DMBA	EGFR	Using western blot and IHC, EGFR signaling was shown to be active in DMBA induced mammary tumors.	[70]
ETV6-Ntrk3	Src	ETV6-Ntrk3 binds to and activates c-Src, and inhibition of c-Src activation blocks EN transforming activity using mouse engineered mouse embryonic fibroblasts.	[71]
Myc	Ras	Activating mutations in K-Ras found in a subset MMTV-Myc induced tumors with a predicted elevation of Ras signalling.	[15]
Myc	B-Catenin	IHC analysis demonstrates higher expression of B-Catenin in the microacinar histology of Myc driven tumors.	[15]
Myc	E2F1 E2F2 E2F3	E2F loss altered tumor latency and Myc proliferative effects on the mammary gland.	[20]
Neu	Akt	Akt loss effects tumor development in the MMTV-Neu mouse model.	[72]
Neu	B-Catenin	Using a beta-gal reporter, ß-catenin/TCF-dependent transcription was shown to be elevated in MMTV-Neu mouse mammary glands.	[73]
Notch	B-Catenin	Knocking down Notch in a human breast cancer cell line also impacted levels of beta-catenin.	[74]
PyMT	Tgfb	Blockade of TGF-beta inhibits mammary tumor metastasis.	[75]
PyMT	Src	Loss of c-Src greatly reduced the occurrence of mammary tumors in the MMTV-PyMT mouse model.	[76]
Tag	Ras	K-ras amplifications observed in large t-antigen mediated tumorigeneis.	[77]
Tag	E2F2 E2F3 RB KO	Large T Antigen simulates loss of Rb by leading to deregulated acitvation of the E2F transcription factors.	[78]
Wnt	p53	MMTV-Wnt1 mammary tumors with mutant p53 exhibited a superior clinical response compared to tumors with wild-type p53.	[79]

DMBA, 7,12-dimethylbenz[a]anthracene; EGFR, epidermal growth factor receptor; IHC, immunohistochemistry; MMTV, mouse mammary tumor virus; PyMT, polyoma middle T; TGF, transforming growth factor.

### Comparisons to human breast cancer

**Figure 5:**
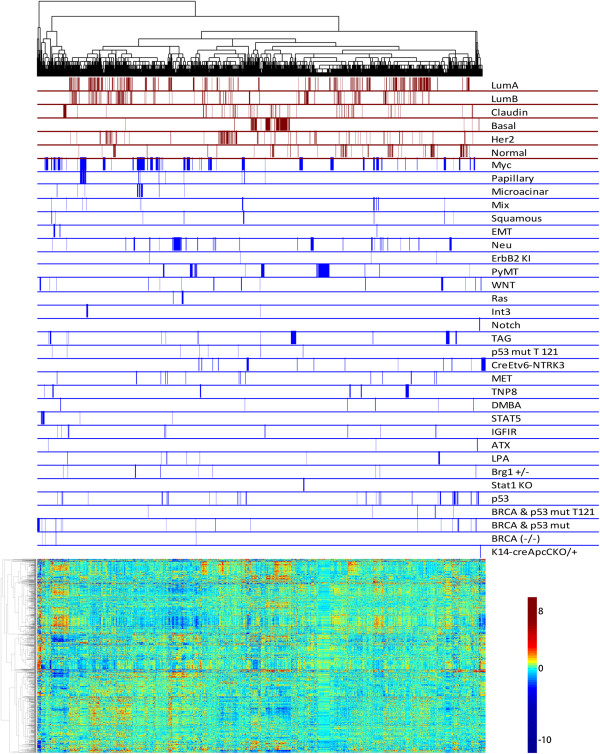
**Unsupervised hierarchical clustering of mouse mammary tumor and human breast cancer gene expression data.** Across the top, the dendrogram illustrates the relationship between human and mouse tumor samples on the basis of gene expression profiles. The red bars mark the intrinsic subtype of each human tumor sample according the annotation on the same line. The blue bars correspond to the mouse mammary tumor type. Below this, a heatmap shows the gene expression patterns for each sample, with expression values illustrated according to the color bar on the right. The dendrogram beside the heatmap shows the correlation between genes based on expression patterns across the samples in the dataset.

With identification of pathways that function in tumor progression in mouse models, it is important to understand whether the given model is reflective of human breast cancer. To this end, we combined datasets for human breast cancer and the mouse mammary tumors in our database, removing both batch and platform effects (Additional file 16). To investigate the relationships between the mouse mammary tumors and human breast tumors, we used unsupervised hierarchical clustering. We identified a large number of mouse mammary tumor models that had similarities in gene expression profiles to human breast cancer (Figure 5). Importantly, Myc and Met induced tumors both recapitulate the heterogeneity observed in human breast cancer. Using histological annotations, specific relationships between Myc tumor types and human breast cancer subtypes were observed (Additional file 17). For example, Myc tumors with an EMT histology clustered together with human claudin low breast cancer. Extending this to the cluster of tumors predicted to have mesenchymal gene expression features (Figure 1C), we observed that a large majority of these tumors also clustered with claudin low breast cancer. Importantly, further investigation of these tumors matched marker expression for claudin low tumors (Additional file 18A-K). Together these data demonstrated that there are mouse models that share human breast cancer heterogeneity with individual tumor types that are closely related to subsets of human breast cancer at the level of gene expression.

**Figure 6:**
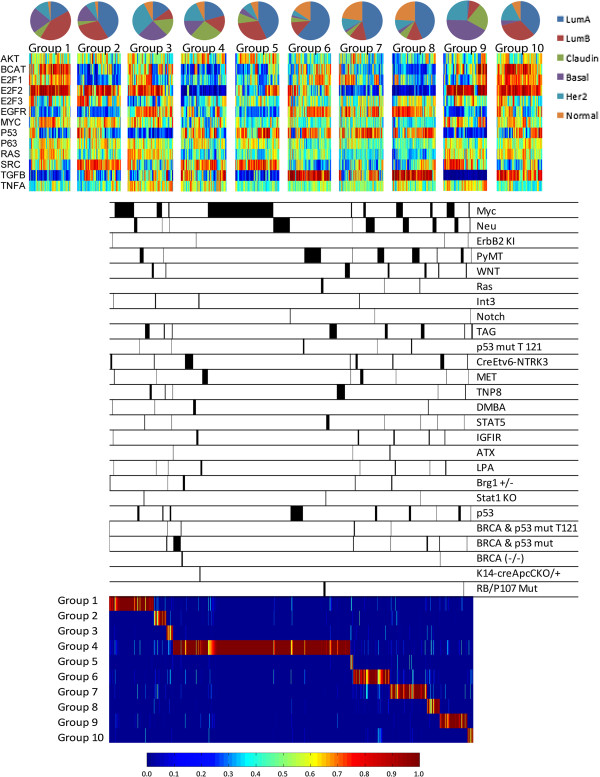
**Mixture modeling analysis of human breast cancer pathway heterogeneity and relationships to mouse models of breast cancer.** Pie charts above each heatmap illustrate the distribution of the intrinsic subtype of samples in each group, according to the color-coded legend. The heatmap for groups 1 to 10 shows predicted pathway activity with probabilities corresponding to the color bar at the bottom of the figure. Below this, black bars mark the samples corresponding to annotations on the same line. Following the samples down to the heatmap below the black bars, the probability that a mouse model has similar pathway activation profiles is shown for each group. Probabilities for this heatmap are shown according to the color bar at the bottom of the figure.

In addition to comparing mouse mammary tumors and human breast cancer with gene expression, we tested relationships using pathway activation predictions. Using a mixture modeling approach, we clustered human breast cancer into ten different groups based on pathway activation profiles (Figure 6). The pie chart above each heatmap shows the spectrum of the intrinsically annotated samples in each group. No single group was made up of one intrinsic subtype, illustrating the heterogeneity of pathway activation within and between intrinsic subtypes of breast cancer. After groups of human tumors were identified, the probability that an individual mouse mammary tumor belonged to a group of human breast cancer was calculated using the pathway activation profile of the mouse mammary tumor sample. Observing these probabilities with a heatmap, we noted that no single group of human breast cancer was modeled by a single mouse mammary tumor type at the pathway level. Instead, for each group of human breast cancer, multiple mouse models showed similar predicted pathway activation profiles. Further, these results demonstrated that mouse model relationships to human breast cancer extended beyond the initiating oncogene. For example, mouse tumors initiated by Myc overexpression contained several different tumor types, each modeling a different group of human breast cancer including those groups that have lower predicted Myc activity. Moreover, Neu initiated tumors using an inducible promoter frequently model a single group of human breast cancer (Additional file 19), while other Neu models have diverse pathway activation profiles leading to relationships with several different groups of human breast cancer. These results considered together highlight the similarity and differences between mouse models and human breast cancers.

## Discussion

Here we have described the genomic analysis of a dataset composed of publicly available gene expression data for mouse models of breast cancer. These data have been analyzed through a variety of mechanisms to ask how mouse models are distinct, what properties they share and how they reflect human breast cancer. These data indicate that great care should be taken to appropriately choose the mouse model to use and that a genomic and histological characterization of tumors should be completed following experimentation.

In the examination of mouse models in the database, unsupervised hierarchical clustering revealed significant heterogeneity both between models and within models and was pronounced in tumor models with a large number of samples. Between model differences were fully expected given the unique initiating events causing tumor formation. However, prior studies with relatively few samples for each model did not demonstrate extensive within model heterogeneity [18]. In comparison, we have demonstrated extensive heterogeneity within many models. In part this is due to differences between intrinsic clustering methods [80] and unsupervised hierarchical clustering. However, given that we have noted corresponding differences in fold change, GSEA predictions and pathway signature probabilities, it is likely that this is a reflection of the number of samples used in the analysis. As such, this provides an important caution to characterize a sufficiently large population of tumors to capture heterogeneity in the analysis.

Given that there is typically a predominant histological pattern associated with a given GEM tumor type [81], it is not surprising that there is a predominant genomic pattern. Indeed, we noted for many models that histology is predictive of the genomic subtype. Interestingly, this histological and genomic interaction is capable of spanning tumor initiating events from different mouse models. Indeed, EMT and spindle-type tumors from diverse models clustered together and were distinct from the non-EMT samples originating in the same model system. Thus, it is also critical for investigators to analyze all tumors from a given model for both histological and genomic patterns.

Mouse models were also investigated individually in comparison to the entire dataset using a variety of methods. This revealed characteristic gene expression patterns at the fold change level, specific GSEA enrichment effects and key pathway signature differences. In many cases, these results correlated with prior studies. For instance, annotation of fold change results predicted that Neu induced tumors upregulated Krox 20 which is consistent with previous chromatin immunoprecipitation (ChIP) results [82]. When pathway signatures were examined, there were a large number of predictions that could be made for pathways used in specific GEM tumor models. Importantly, while these pathway signatures have previously been validated [2], the model by model pathway predictions shown in Table 2 are highly consistent with previously published tests. For instance, the pathway signatures predicted a high probability of Src activation in PyMT tumors in the FVB background and recent work has demonstrated the necessity for c-Src in PyMT induced tumors [76]. Collectively, for the pathways listed in Table 2, we note agreement between the pathway signature predictions and the reported genetic crosses. Moreover, the pathway signature predictions are also reflective of additional mutations that accumulate in the samples. This was noted in the Myc and TAG induced tumors where the Ras signature was predicted to be elevated, consistent with the large number of Ras activating mutations in these strains [15,77]. Given that numerous published genetic tests are in agreement with the pathway predictions, the remaining cell signaling pathway predictions offer a large number of testable hypotheses. In the future, pathway predictions in the various models should prove to be an important resource for initiating studies into investigating the importance of various signaling pathways in tumor biology.

One of the key aspects of this study was the comparison between mouse models and human breast cancer. These data demonstrated similarities and differences between the two groups and should serve as an important consideration when attempting to extend the comparison of mouse models to human cancer. Taking into account the clustering data, we readily noted that the heterogeneity between human breast cancer samples was present within individual mouse models. Despite capturing the genomic diversity of the samples, we noted several samples with no genomic similarity to human breast cancer, including tumors from strains with other samples that had clear similarity to human breast cancer. This clearly suggests that if conclusions are to be drawn from mouse models of breast cancer, that the mouse samples should be compared and clustered with a variety of human tumors.

In addition to clustering of genomic data, we compared mouse models to human breast cancer through signaling pathway activation predictions. These results showed that for any given group of human breast cancer samples, there was a mouse model with similar pathway activation profiles. Using these results, it is possible to select the mouse model that most closely represents a group of human breast cancer for the signaling pathways of interest. However, it is critical to consider both clustering and pathway activation and to combine these methods to choose the most appropriate model to mimic human breast cancer. For example, to model HER2+ breast cancer and to study the role of HER2 in tumor development, research initially used the MMTV-Neu mice [7]. However, the gene expression data reveals that this strain does not associate with the HER2+ human samples through genomic clustering. However, mixture modeling indicated that a proportion of HER + human cancers did group with the MMTV-Neu samples at the level of pathway activation. This indicates that in some aspects the mouse model is appropriately related to human HER2+ breast cancer. Further, recent reports demonstrate that a strain of mice with conditional activation of Neu under the control of the endogenous promoter which undergo amplification [8] far more closely recapitulate human HER2+ breast cancer [21]. Taken together, these data illustrate the importance of fully characterizing and using all genomic information to select the appropriate model for examination.

Recent reports have described the development of serially transplantable human breast cancer samples that are grown in a murine host with clear genomic similarity to the primary human breast cancer samples [83] and obviously this is an optimal model for specific studies. However, there is clear utility for GEM models, especially with regard to the ability to ask defined genetic questions with regard to key signaling pathways in tumor biology. As such, the prior characterization of mouse and human breast cancer similarities was a critical development [18]. The expanded number of samples and methods of analysis in this report have clearly illustrated additional components of mouse breast cancer biology that require careful consideration. Indeed, the extent of genomic heterogeneity was only appreciated previously for select models [11,15-17], but our work indicates that this is a general characteristic across the majority of breast cancer model systems. As such, this work underscores the requirement to fully characterize mouse tumor biology at histological and genomic levels before a valid comparison to human breast cancer may be drawn. Thus, we have provided the complete files for all of the comparisons made in this manuscript, from fold change between models to GSEA and pathway predictions, with the intent of this being used as a resource to choose and compare mouse models in breast cancer research.

## Conclusions

Collectively, our work demonstrates genomic heterogeneity in mouse mammary tumor models. As an additional outcome of this research, we have provided a large scale predictive resource for each of the mouse models in the database. With heterogeneity driving a variety of relationships between individual mouse mammary tumors and human breast cancer, this work highlights the necessity of fully characterizing mouse tumor biology at molecular, histological and genomic levels before a valid comparison to human breast cancer may be drawn.

## Additional files

1, 2, 3, 6, 7, 11, 12 are available for download at: https://www.msu.edu/~andrech1/BCR_Supplemental/BCR_Supplemental.html. The results of each analysis are provided as links to zipped folders as described below and are numbered according to their reference in the manuscript. Clicking on a link will begin the download of the zipped material.

## Abbreviations

DMBA: 7,12-dimethylbenz[a]anthracene; EMT: epithelial to mesenchymal transition; ENCODE: Encyclopedia of DNA Elements; GATHER: gene annotation tool to help explain relationships; GEM: genetically engineered mice; GSEA: gene set enrichment analysis; HER2: human epidermal growth factor receptor 2; MMTV: mouse mammary tumor virus; PCA: principle components analysis; PyMT: polyoma middle T.; SAM: significance analysis of microarrays; TAG: large T antigen; TCA: the citric acid cycle; TCGA: The Cancer Genome Atlas; TNF: tumor necrosis factor; TRANSFAC: transcription factor database.

## Competing interests

The authors declare that they have no competing interests.

## Authors’ contributions

DH and EA conceived of the study, and participated in its design and coordination and helped to draft the manuscript. DH performed the experiments in this study. DH and EA interpreted the data. Both authors read and approved the final manuscript.

## Supplementary Material

Additional file 1 PCA code for Matlab. Click here for file

Additional file 2 Fold change values organized by mouse tumor model type comparing the mouse model to all other types of mouse models. Click here for file

Additional file 3 Fold change values organized by mouse tumor model type comparing the mouse model to normal mammary gland. Click here for file

Additional file 4: Table S1 **Gene signature settings for pathway predictions.** Specific settings are shown for each gene signature of pathway activation. Click here for file

Additional file 5: Figure S1 Removal of Batch Effects from Affymetrix Datasets. (A) Affymetrix datasets color coded according to the study of origin in a principle components analysis plot prior to BFRM batch effect correction. (B) Affymetrix datasets color coded according to the study of origin in a principle components analysis plot after BFRM batch effect correction. (C) Affymetrix datasets are color coded together in blue after BFRM batch effect correction. The various Agilent gene expression datasets are color-coded and plotted along with Affymetrix data on the three principle components to illustrate platform and batch variance. (D) Agilent and Affymetrix color-coded data plotted after COMBAT removed batch and platform technical variance. (E) Neu-induced tumors are color coded in blue and all other tumors are in green, illustrating variance between similar tumor types on the basis of platform and batch artifacts. (F) Neu-induced tumors are color coded in blue and all other tumors are in green illustrating mediation of batch and platform effects. Click here for file

Additional file 6 Fold change for genes in clusters 1-4 from the manuscript and the gene ontology associated with each cluster. Click here for file

Additional file 7 GSEA for clusters 1-4, each of the runs (C2, C3, and so on) is in a separate folder. Click here for file

Additional file 8: Figure S2 Gene set enrichment analysis for mouse mammary tumors in the black color-coded cluster. (A) A gene set for down regulated genes in mesenchymal breast cancer is significantly enriched (*P* <.0001) and down regulated in the black cluster (cluster4) of tumors. (B) A gene set for Zeb1 target genes is significantly enriched (*P* = .005) for low expression for the tumors in the black cluster. (C) A gene set for genes highly expressed in mammary stem cells is significantly enriched (*P* = .016) and upregulated in tumors from cluster 4 (black). (D) A gene set for genes that are down regulated in mammary stem cells is significantly enriched (*P* <.0001) and also down regulated in the cluster 4 (black) tumors. Click here for file

Additional file 9: Figure S3 Tumors that were classified for mesenchymal histology cluster into the black cluster. Highlighting prior histological annotations for mesenchymal or EMT-like tumors across the Myc, IGF-IR, DMBA, and p53 mutant models show that a large majority of these tumors cluster together in the black cluster. Click here for file

Additional file 10: Figure S4 Gene set enrichment analysis for mammary cell types across major clusters of mouse mammary tumors. GSEA for tumors in blue cluster compared to all other clusters show significant enrichment for a mammary luminal progenitor cell gene expression signature (*P* = .006). Similarly, tumors from the green cluster associate with a mixture of luminal cell gene expression features, while tumors in the orange cluster are significantly enriched for gene expression features of mature luminal cells (*P* = .04). Lastly, tumors in the black cluster are significantly enriched for gene expression features of mammary stem cells (*P* = .01). Click here for file

Additional file 11 **GSEA for mouse models compared to all other models or to mammary gland development.** Listed by model. Click here for file

Additional file 12 PDFs of pathway predictions for each mouse model of breast cancer, folders exist for each mouse modelx. Click here for file

Additional file 13: Figure S5 Unsupervised hierarchical clustering of pathway probabilities for PyMT induced tumors. The dendrogram across the top illustrates the relationship between PyMT tumor types on the basis of pathway activation profiles. Below the dendrogram black bars correspond to sample details on the same line, annotating the genetic background and sample type for each sample. The heatmap shows the predicted pathway activity according to the probabilities listed on the color bar below the heatmap. Directly beside the heatmap, a vertical dendrogram illustrates the degree of correlation between pathways across the samples. Click here for file

Additional file 14: Figure S6 Unsupervised hierarchical clustering of pathway probabilities for Myc induced tumors. The dendrogram across the top illustrates the relationship between Myc tumor types on the basis of pathway activation profiles. Below the dendrogram black bars correspond to sample details on the same line, annotating the tumor histology (if known), specific form of Myc expression, recurrence status, and additional modifications. The heatmap shows the predicted pathway activity according to the probabilities listed on the color bar below the heatmap. Directly beside the heatmap, a vertical dendrogram illustrates the degree of correlation between pathways across the samples. Click here for file

Additional file 15: Figure S7 Unsupervised hierarchical clustering of pathway probabilities for Neu induced tumors. The dendrogram across the top illustrates the relationship between Neu tumor types on the basis of pathway activation profiles. Below the dendrogram black bars correspond to sample details on the same line, annotating the specific form of Neu, and additional modifications. The heatmap shows the predicted pathway activity according to the probabilities listed on the color bar below the heatmap. Directly beside the heatmap, a vertical dendrogram illustrates the degree of correlation between pathways across the samples. Click here for file

Additional file 16: Figure S8 Removal of batch effects between mouse and human breast cancer datasets. (A) Mouse (blue) and human (green) Affymetrix data gene expression variance plotted onto three principle components prior to BFRM. (B) Mouse (blue) and human (green) Affymetrix data gene expression variance plotted onto three principle components after BFRM. (C) Human(green) and mouse (blue) Affymetrix data after BFRM correction put together with mouse Agilent data (red) prior to COMBAT. (D) Human(green) and mouse (blue) Affymetrix data after BFRM correction put together with mouse Agilent data (red) after COMBAT artifact correction. Click here for file

Additional file 17: Figure S9 Unsupervised hierarchical clustering of Myc mouse mammary tumors and human breast cancer gene expression data. Across the top, the dendrogram illustrates the relationship between human and mouse tumor samples on the basis of gene expression profiles. The red bars mark the intrinsic subtype of each human tumor sample according to the annotation on the same line. The blue bars correspond to the Myc mouse mammary tumor type. Below this, a heatmap shows the gene expression patterns for each sample, with expression values illustrated according to the color bar on the right. The dendrogram beside the heatmap shows the correlation between genes based on expression patterns across the samples in the dataset. Click here for file

Additional file 18: Figure S10 Claudin low marker expression in the black cluster mouse mammary tumors. Claudin low marker expression comparisons for cluster 4 (black) tumors compared to tumors in all other clusters as defined by Figure 1A. (A-C) Cell adhesion markers that have low expression in claudin low human tumors are also down regulated in cluster 4 (black tumors), *P* <.0001. (D-E) Genes that are involved with the immune system that are found to be highly expressed in claudin low human tumors are highly expressed in mouse cluster 4 tumors (black), *P* <.01 for CD79B and *P* <.0001 for VAV1. (F) Chemokine (C-X-C motif) ligand 12, involved in cell communication and previously shown to be highly expressed in claudin low tumors, is upregulated in cluster 4 (black) mouse mammary tumors, *P* <.0001. (G) Fibroblast growth factor 7, an extracellular matrix related factor and previously shown to be highly expressed in claudin low tumors, is upregulated in cluster 4 (black) mouse mammary tumors, *P* <.0001. (H-J) Cell migration markers previously shown to be highly expressed in human claudin low tumors are upregulated in mouse cluster 4(black) tumors, *P* <.02 for moesin and *P* <.0001 for integrin α5. (K) Angiogenesis marker, VEGFC, was previously shown to be upregulated in human claudin low tumors and is highly expressed in mouse cluster 4(black) tumors. Click here for file

Additional file 19: Figure S11 Mixture modeling highlighting pathway relationships between human breast cancer and specific models of Neu mediated tumorigenesis. Pie charts above each heatmap illustrate the distribution of the intrinsic subtype of samples in each group, according to the color-coded legend. The heatmap for groups 1 to 10 show predicted pathway activity with probabilities corresponding to the color bar at the bottom of the figure. Below this, blue bars mark the samples corresponding to annotations on the same line. Following the samples down to the heatmap below the blue bars, the probability that a specific type of Neu model has similar pathway activation profiles is shown for each group. Probabilities for this heatmap are shown according to the color bar at the bottom of the figure. Click here for file
