# Supplementary material for: A genomic analysis of mouse models of breast cancer reveals molecular features of mouse models and relationships to human breast cancer
Source: Breast Cancer Res. 2014 Jun 5;16(3):R59. doi: 10.1186/bcr3672 (PMC4078930; doi:10.1186/bcr3672)
Supplement: Additional file 1 — PCA code for Matlab. [file bcr3672-S1.zip › AdditionalFile1/Additional_File_1.docx]

Matlab code for PCA generation:

load('name.txt')

x=name

s=svd(x)

[u,s,v]=svds(x);

y=u(:,1:3)

a=x`*y

r=a(:,1)

t=a(:,2)

v=a(:,3)

s=30*ones(45,1);

scatter3(r,t,v,s,c,'filled')
