# Supplementary material for: A genomic analysis of mouse models of breast cancer reveals molecular features of mouse models and relationships to human breast cancer
Source: Breast Cancer Res. 2014 Jun 5;16(3):R59. doi: 10.1186/bcr3672 (PMC4078930; doi:10.1186/bcr3672)
Supplement: Additional file 3 — Fold change values organized by mouse tumor model type comparing the mouse model to normal mammary gland. [file bcr3672-S3.zip › Additional_File_3/STAT1KO_VS_nmlmammary/AdditionalFile79_sTAT1KO_VS_nmlmammary_NA/Note.docx]

No Corresponding genetic background for NML mammary gland
