# Supplementary figures and images for: A genomic analysis of mouse models of breast cancer reveals molecular features of mouse models and relationships to human breast cancer
Source: Breast Cancer Res. 2014 Jun 5;16(3):R59. doi: 10.1186/bcr3672 (PMC4078930; doi:10.1186/bcr3672)

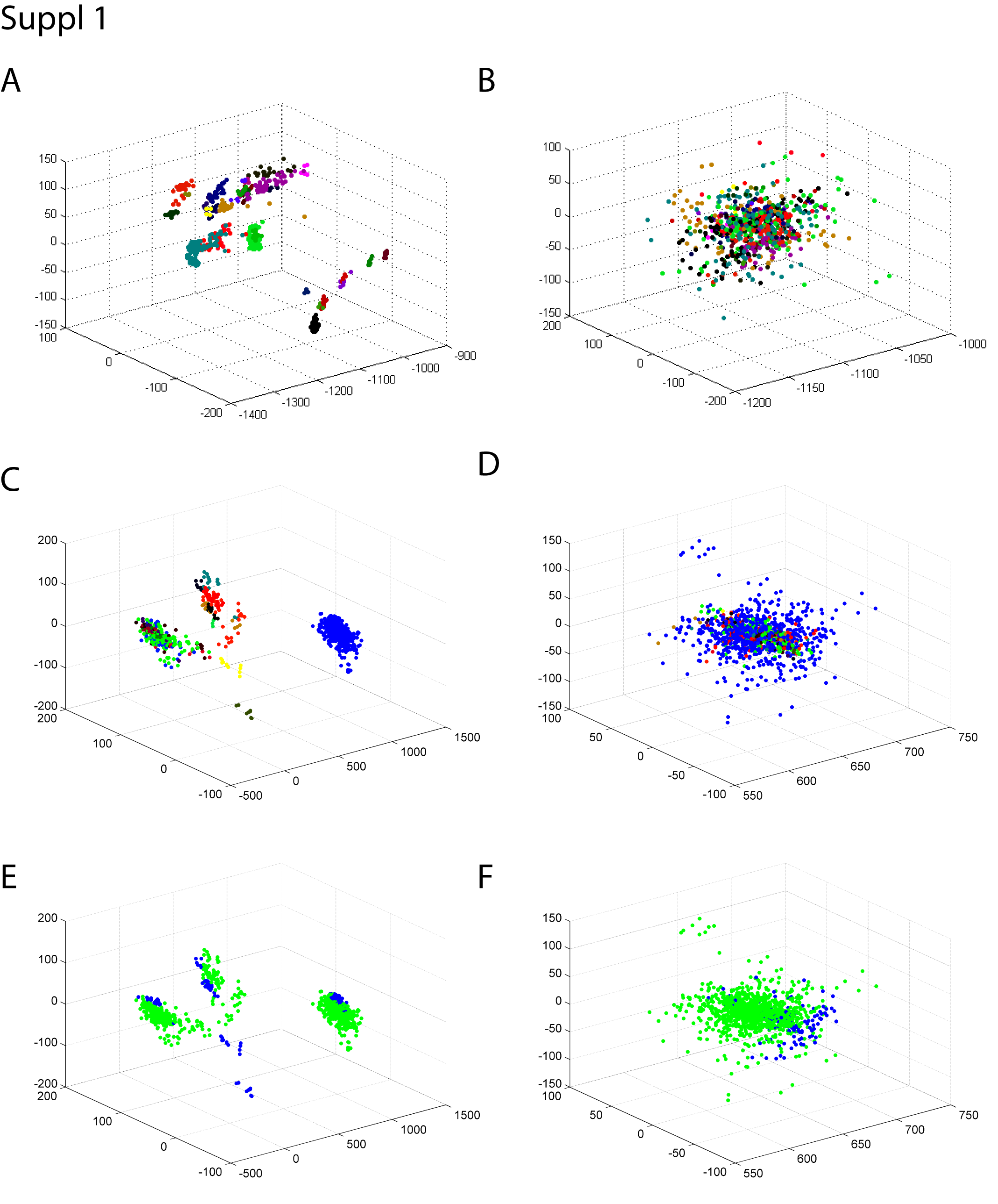

Supplement: Additional file 5: Figure S1 — Removal of Batch Effects from Affymetrix Datasets. (A) Affymetrix datasets color coded according to the study of origin in a principle components analysis plot prior to BFRM batch effect correction. (B) Affymetrix datasets color coded according to the study of origin in a principle components analysis plot after BFRM batch effect correction. (C) Affymetrix datasets are color coded together in blue after BFRM batch effect correction. The various Agilent gene expression datasets are color-coded and plotted along with Affymetrix data on the three principle components to illustrate platform and batch variance. (D) Agilent and Affymetrix color-coded data plotted after COMBAT removed batch and platform technical variance. (E) Neu-induced tumors are color coded in blue and all other tumors are in green, illustrating variance between similar tumor types on the basis of platform and batch artifacts. (F) Neu-induced tumors are color coded in blue and all other tumors are in green illustrating mediation of batch and platform effects. [file bcr3672-S5.tiff]

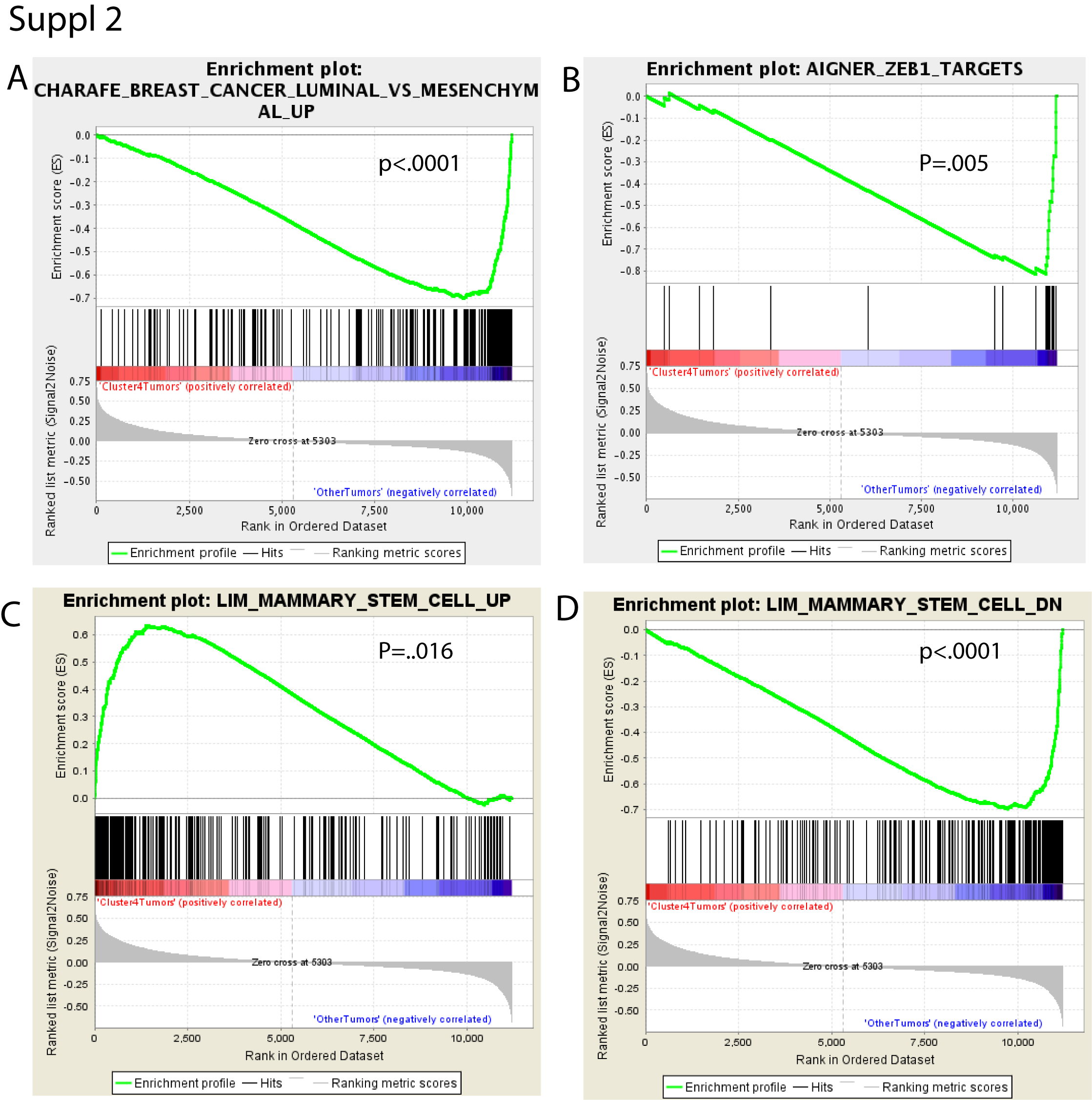

Supplement: Additional file 8: Figure S2 — Gene set enrichment analysis for mouse mammary tumors in the black color-coded cluster. (A) A gene set for down regulated genes in mesenchymal breast cancer is significantly enriched (P <.0001) and down regulated in the black cluster (cluster4) of tumors. (B) A gene set for Zeb1 target genes is significantly enriched (P = .005) for low expression for the tumors in the black cluster. (C) A gene set for genes highly expressed in mammary stem cells is significantly enriched (P = .016) and upregulated in tumors from cluster 4 (black). (D) A gene set for genes that are down regulated in mammary stem cells is significantly enriched (P <.0001) and also down regulated in the cluster 4 (black) tumors. [file bcr3672-S8.tiff]

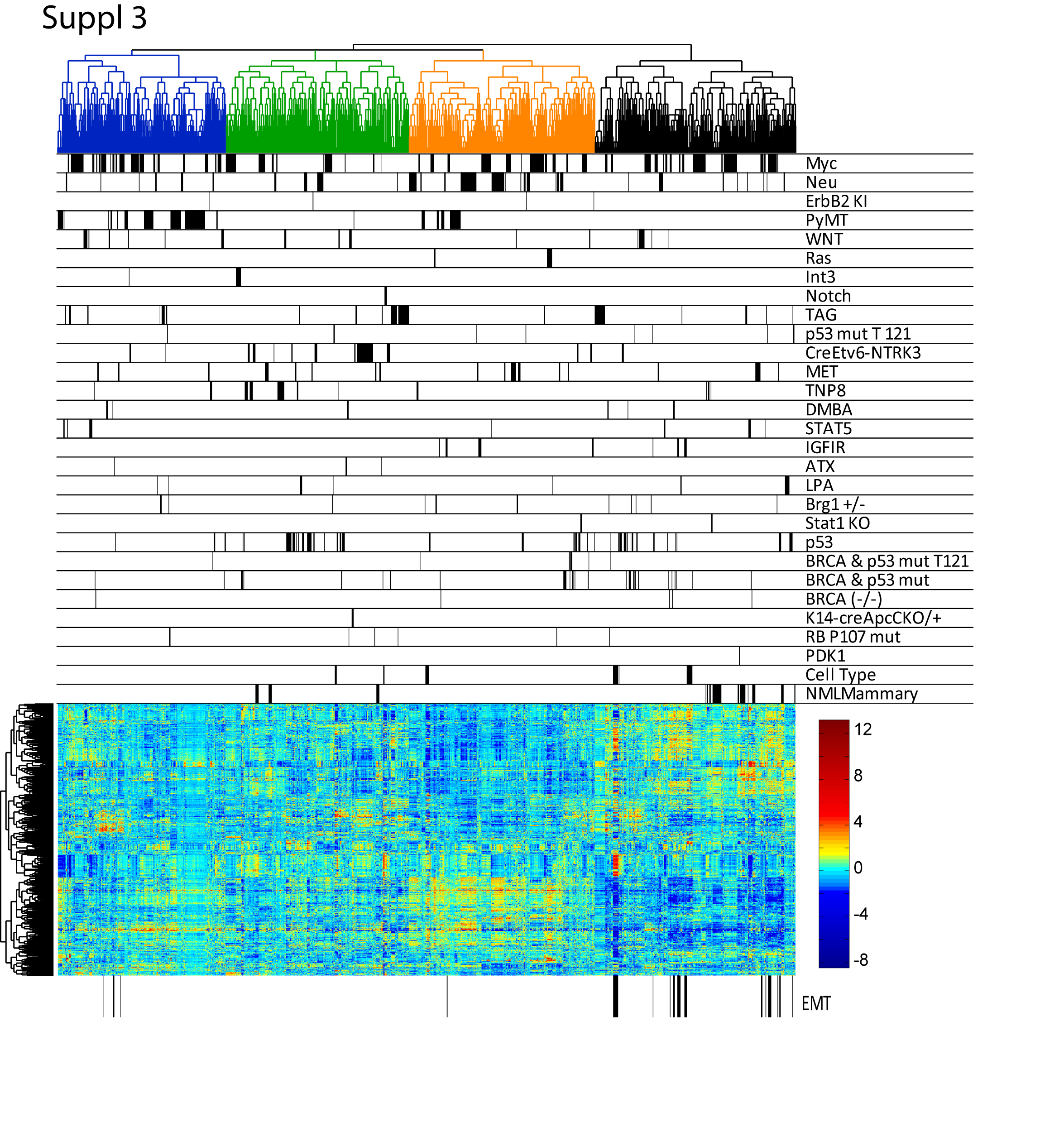

Supplement: Additional file 9: Figure S3 — Tumors that were classified for mesenchymal histology cluster into the black cluster. Highlighting prior histological annotations for mesenchymal or EMT-like tumors across the Myc, IGF-IR, DMBA, and p53 mutant models show that a large majority of these tumors cluster together in the black cluster. [file bcr3672-S9.tiff]

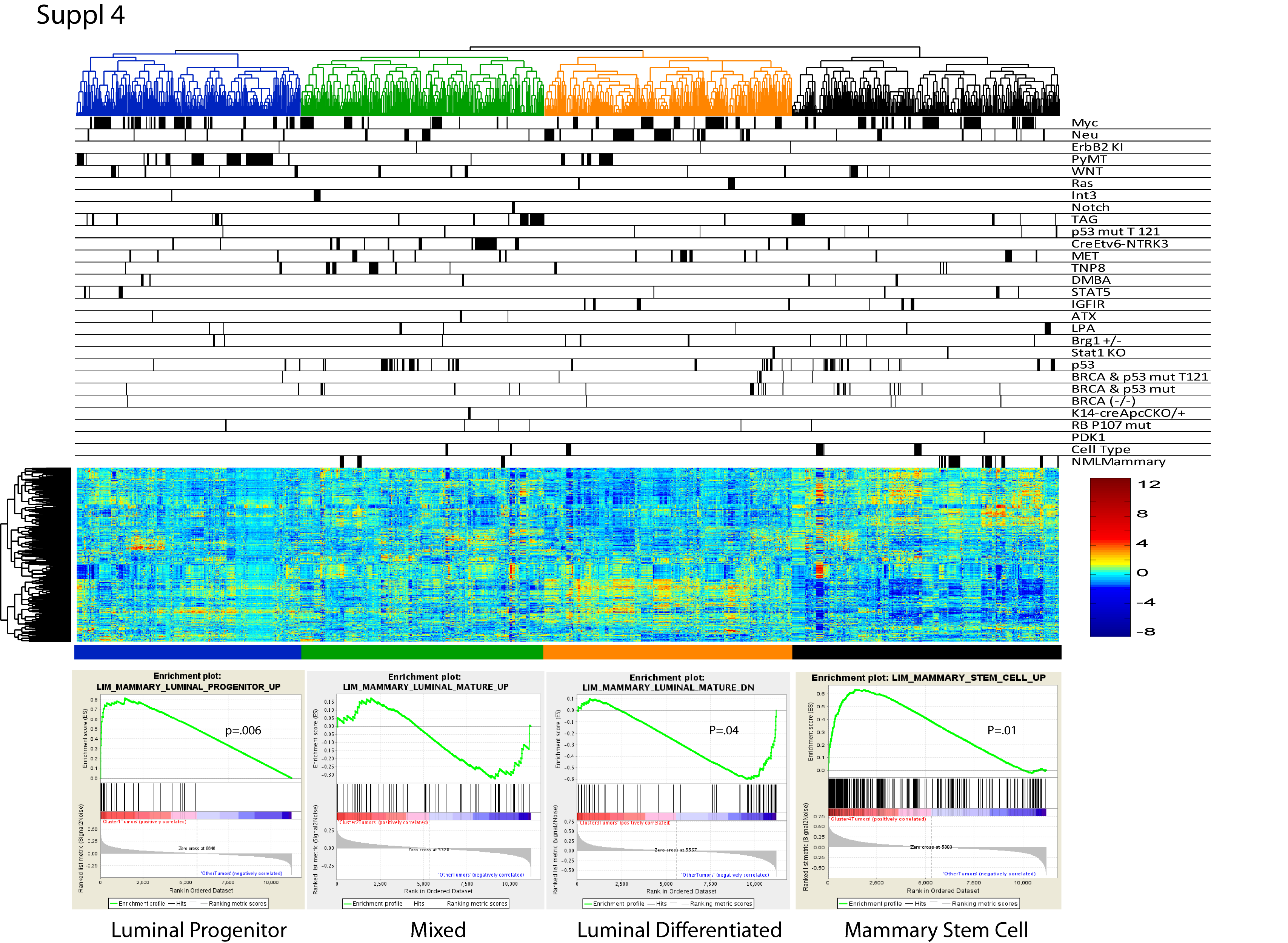

Supplement: Additional file 10: Figure S4 — Gene set enrichment analysis for mammary cell types across major clusters of mouse mammary tumors. GSEA for tumors in blue cluster compared to all other clusters show significant enrichment for a mammary luminal progenitor cell gene expression signature (P = .006). Similarly, tumors from the green cluster associate with a mixture of luminal cell gene expression features, while tumors in the orange cluster are significantly enriched for gene expression features of mature luminal cells (P = .04). Lastly, tumors in the black cluster are significantly enriched for gene expression features of mammary stem cells (P = .01). [file bcr3672-S10.tiff]

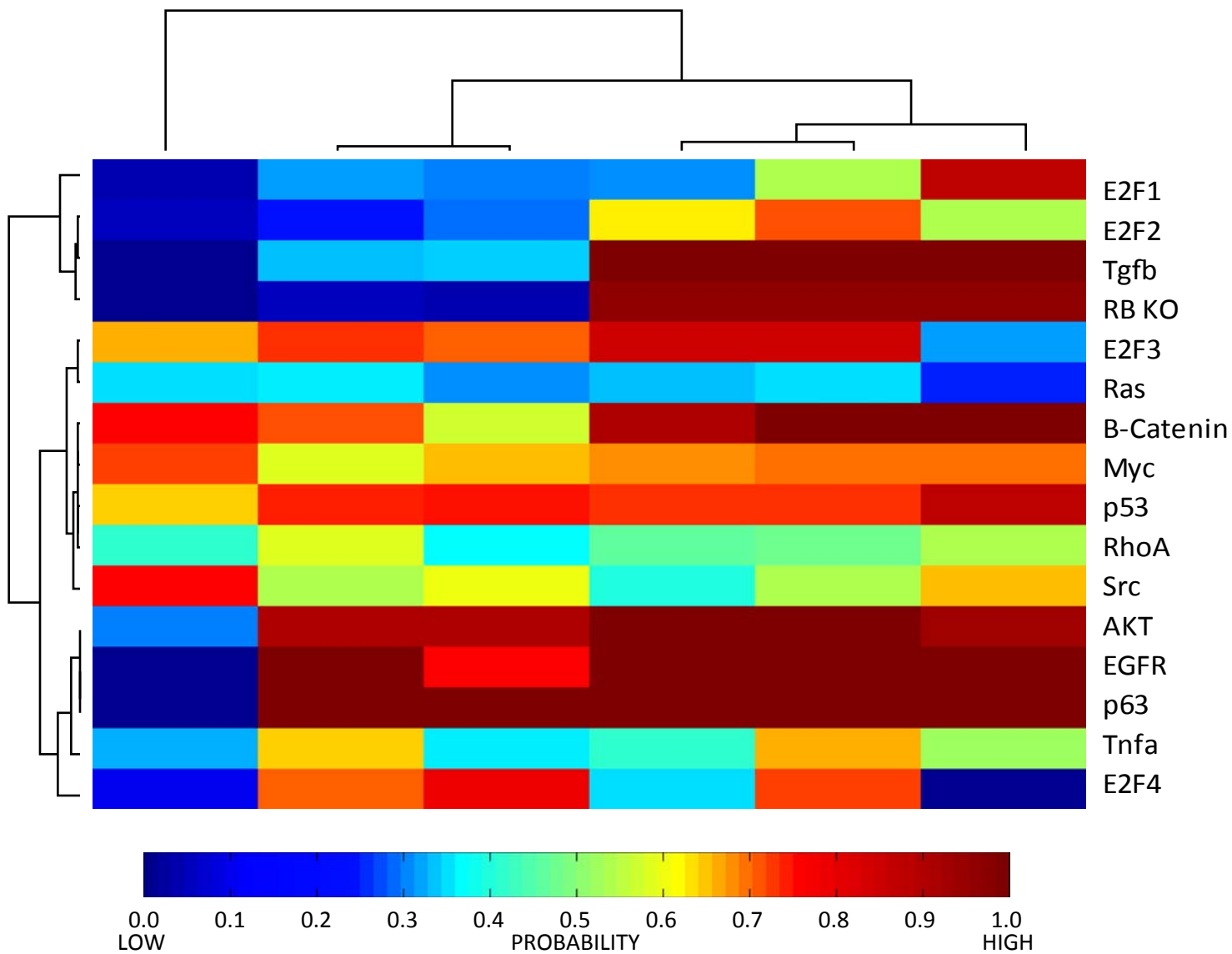

Supplement: Additional file 12 — PDFs of pathway predictions for each mouse model of breast cancer, folders exist for each mouse modelx. [file bcr3672-S12.zip › AdditionalFile12/ApcCKO_pathways/Heatmap.pdf]

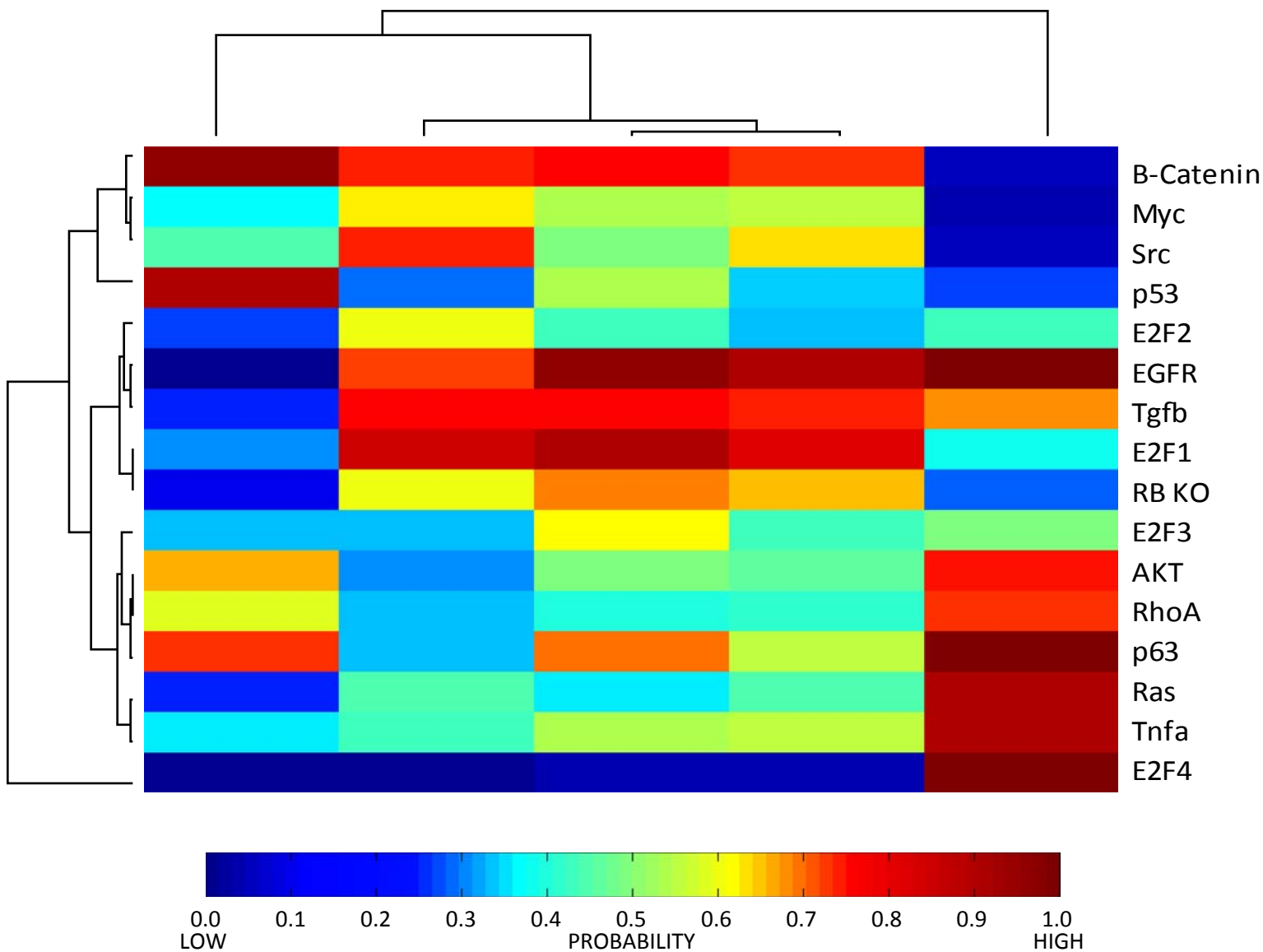

Supplement: Additional file 12 — PDFs of pathway predictions for each mouse model of breast cancer, folders exist for each mouse modelx. [file bcr3672-S12.zip › AdditionalFile12/ATX_pathways/HeatmapATX.pdf]

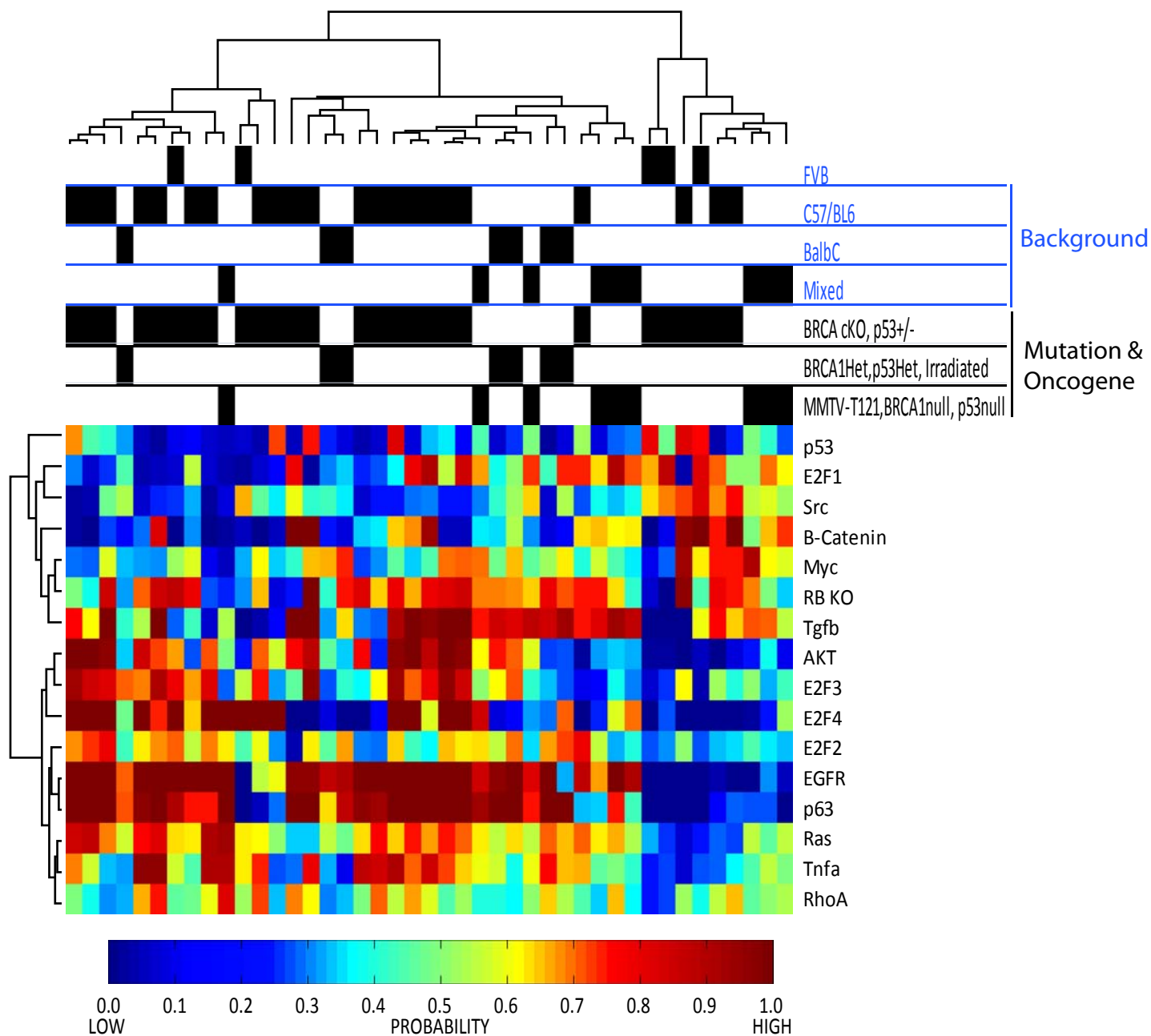

Supplement: Additional file 12 — PDFs of pathway predictions for each mouse model of breast cancer, folders exist for each mouse modelx. [file bcr3672-S12.zip › AdditionalFile12/BRCA_p53_mutant_pathways/Heatmap.pdf]

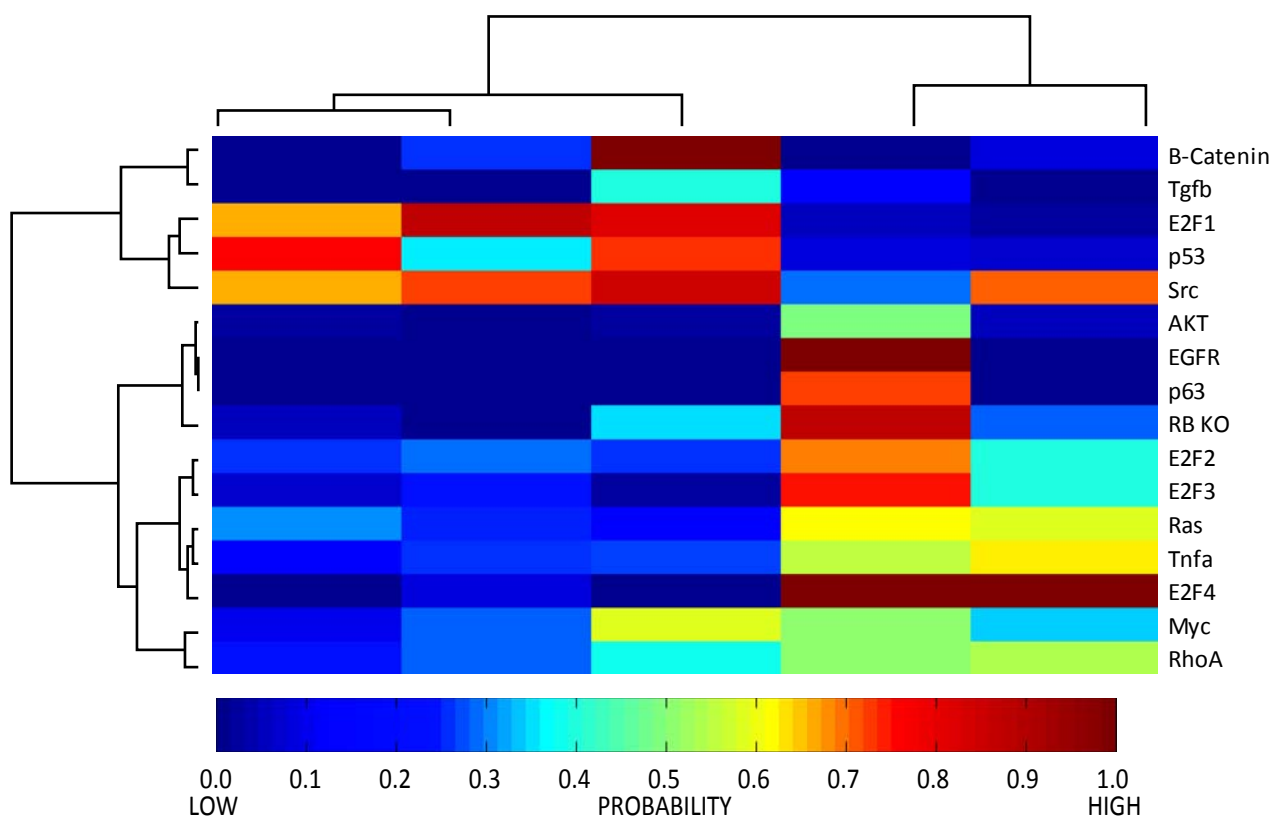

Supplement: Additional file 12 — PDFs of pathway predictions for each mouse model of breast cancer, folders exist for each mouse modelx. [file bcr3672-S12.zip › AdditionalFile12/BRCAnull_pathways/heatmap.pdf]

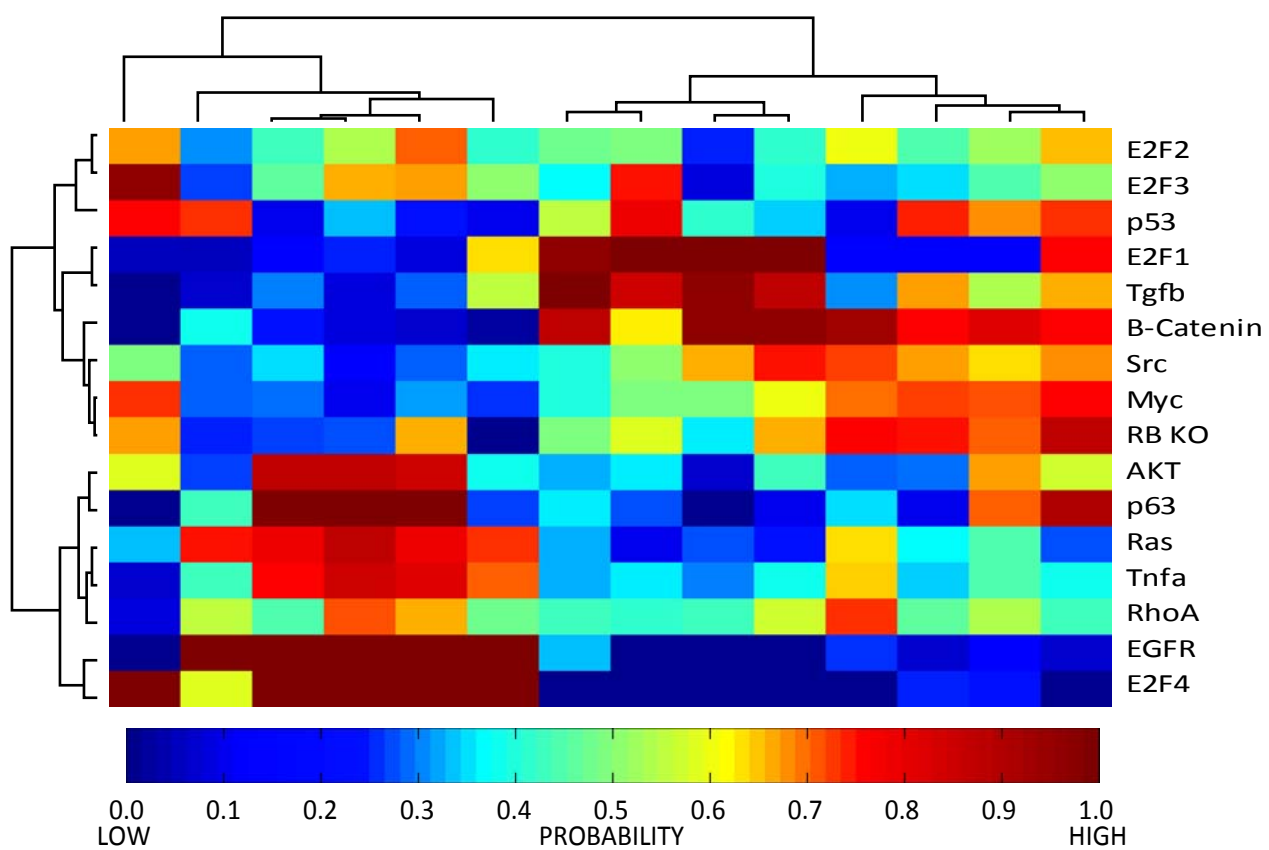

Supplement: Additional file 12 — PDFs of pathway predictions for each mouse model of breast cancer, folders exist for each mouse modelx. [file bcr3672-S12.zip › AdditionalFile12/Brg1Het_pathways/heatmap.pdf]

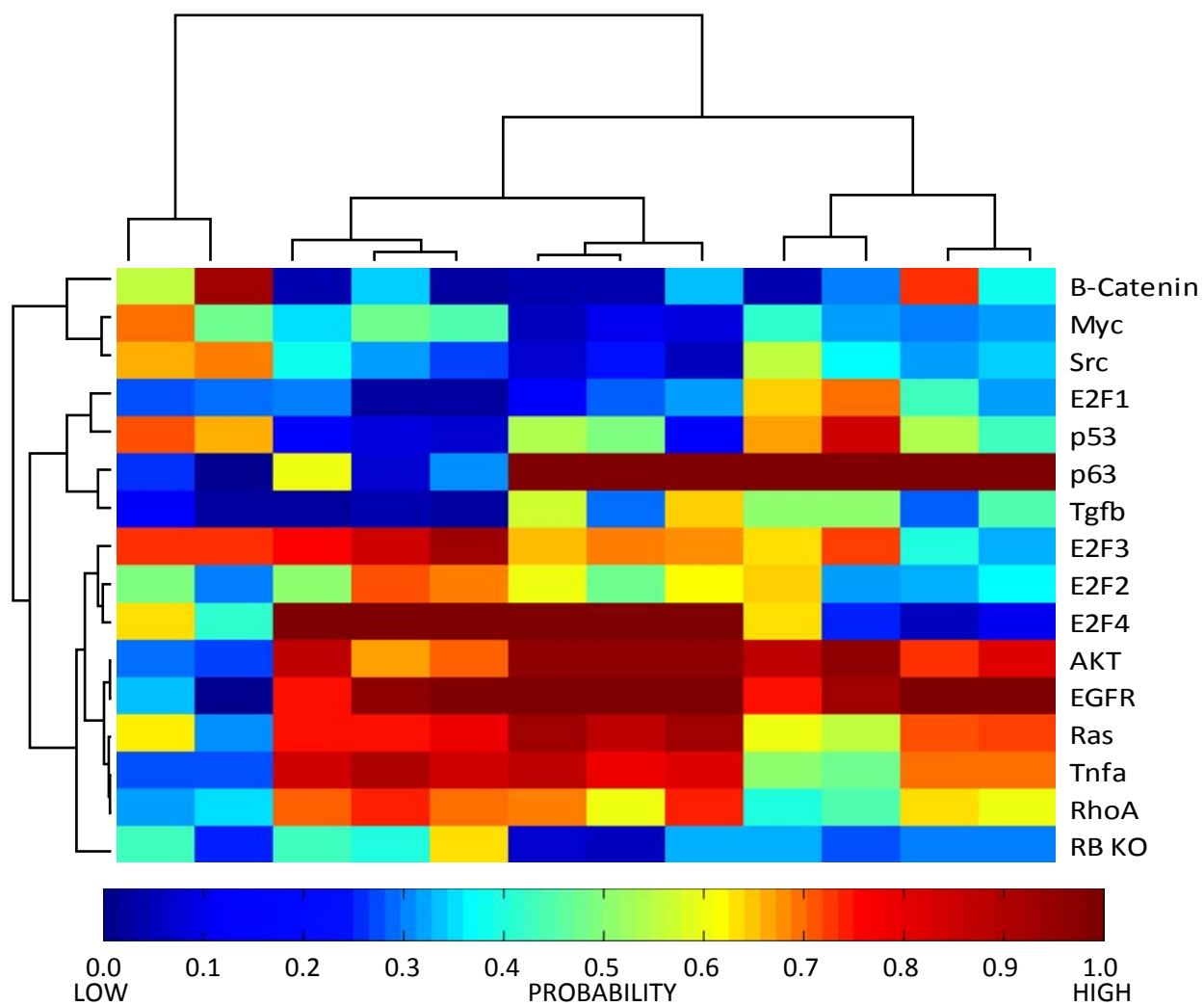

Supplement: Additional file 12 — PDFs of pathway predictions for each mouse model of breast cancer, folders exist for each mouse modelx. [file bcr3672-S12.zip › AdditionalFile12/DMBA_pathways/HeatmapDMBA.pdf]

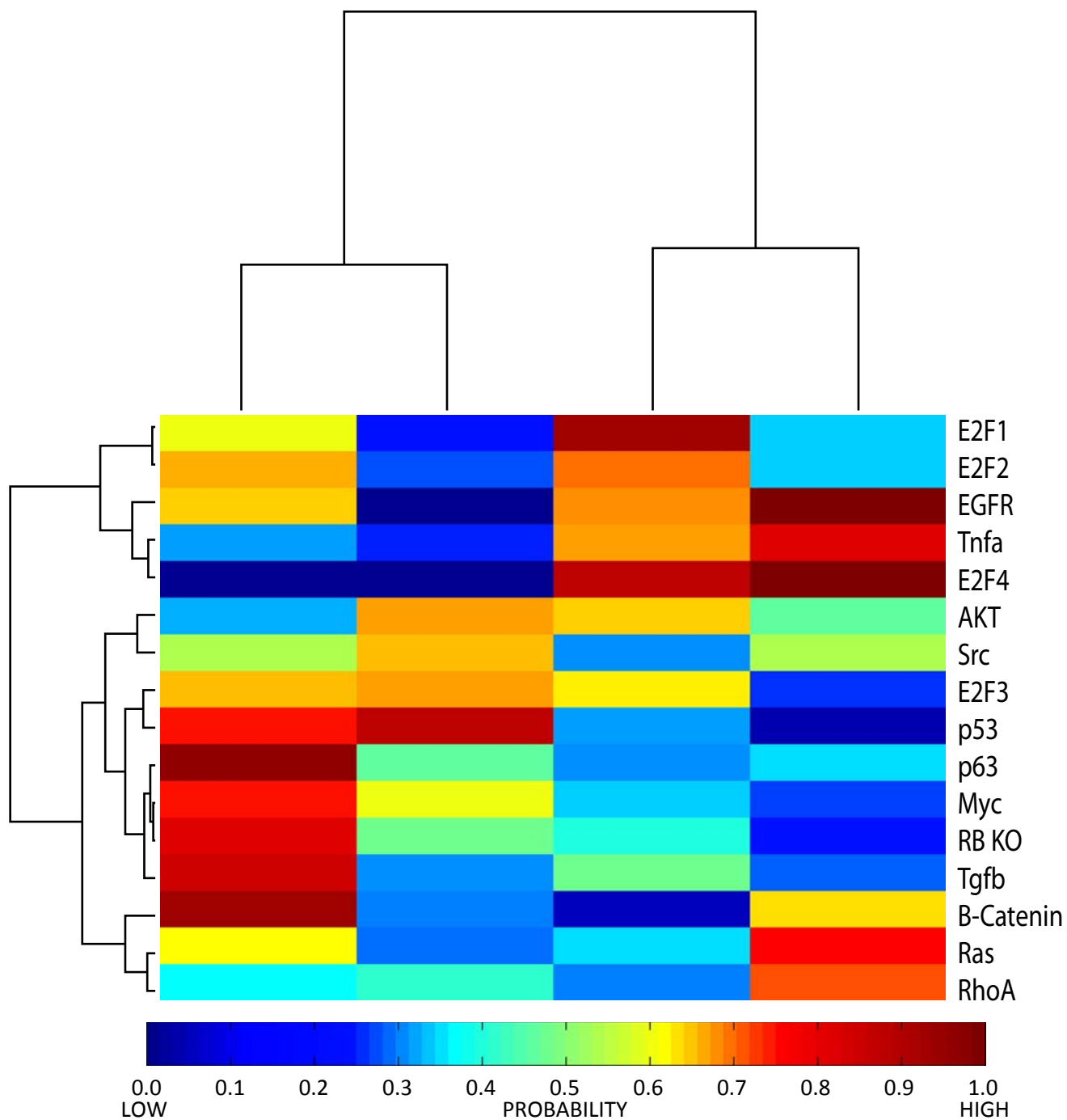

Supplement: Additional file 12 — PDFs of pathway predictions for each mouse model of breast cancer, folders exist for each mouse modelx. [file bcr3672-S12.zip › AdditionalFile12/Erbb2KI_pathways/HeatmapErbb2KI.pdf]

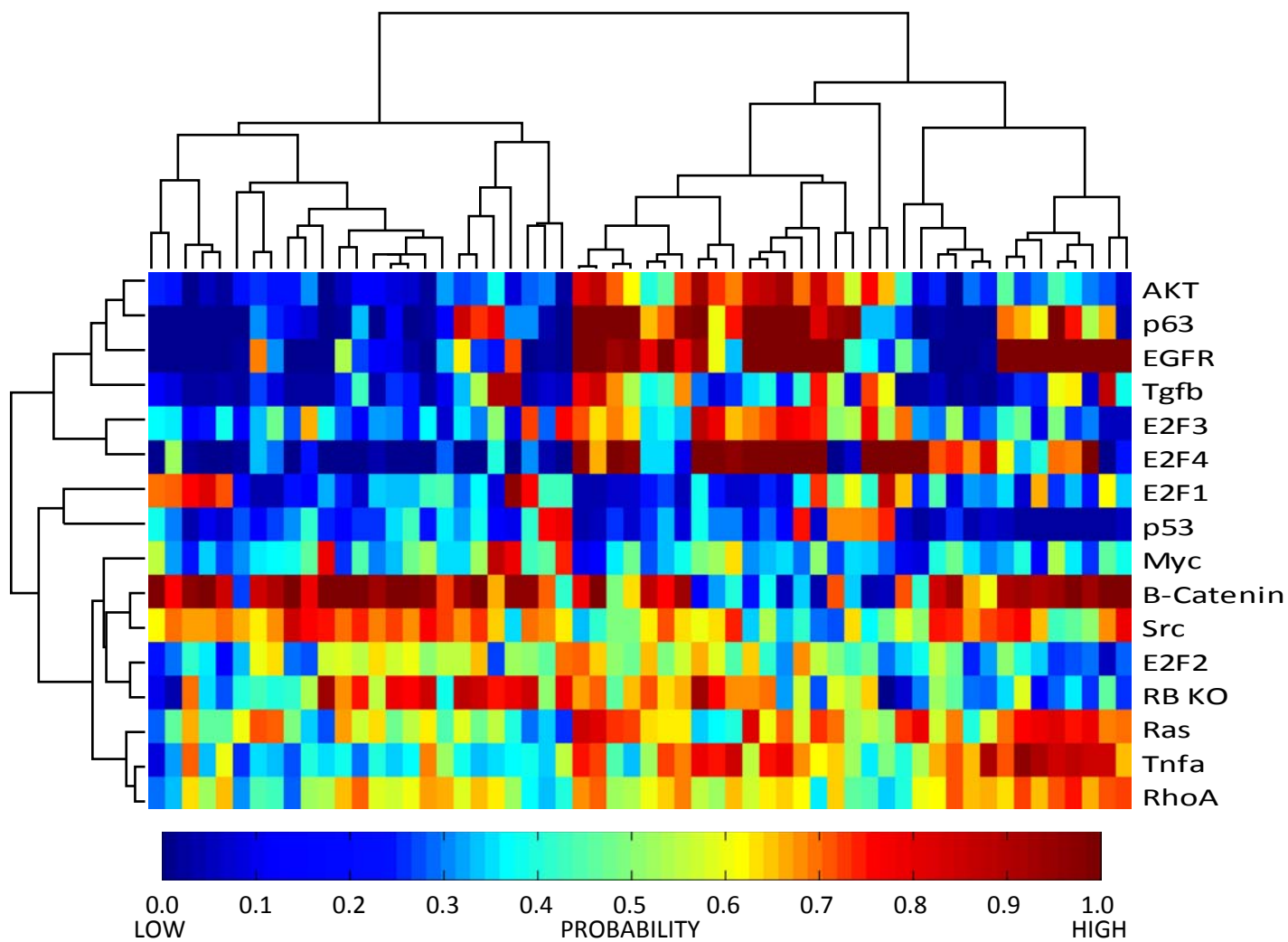

Supplement: Additional file 12 — PDFs of pathway predictions for each mouse model of breast cancer, folders exist for each mouse modelx. [file bcr3672-S12.zip › AdditionalFile12/ETV6Ntrk3_pathways/Heatmap_etv6ntrk3.pdf]

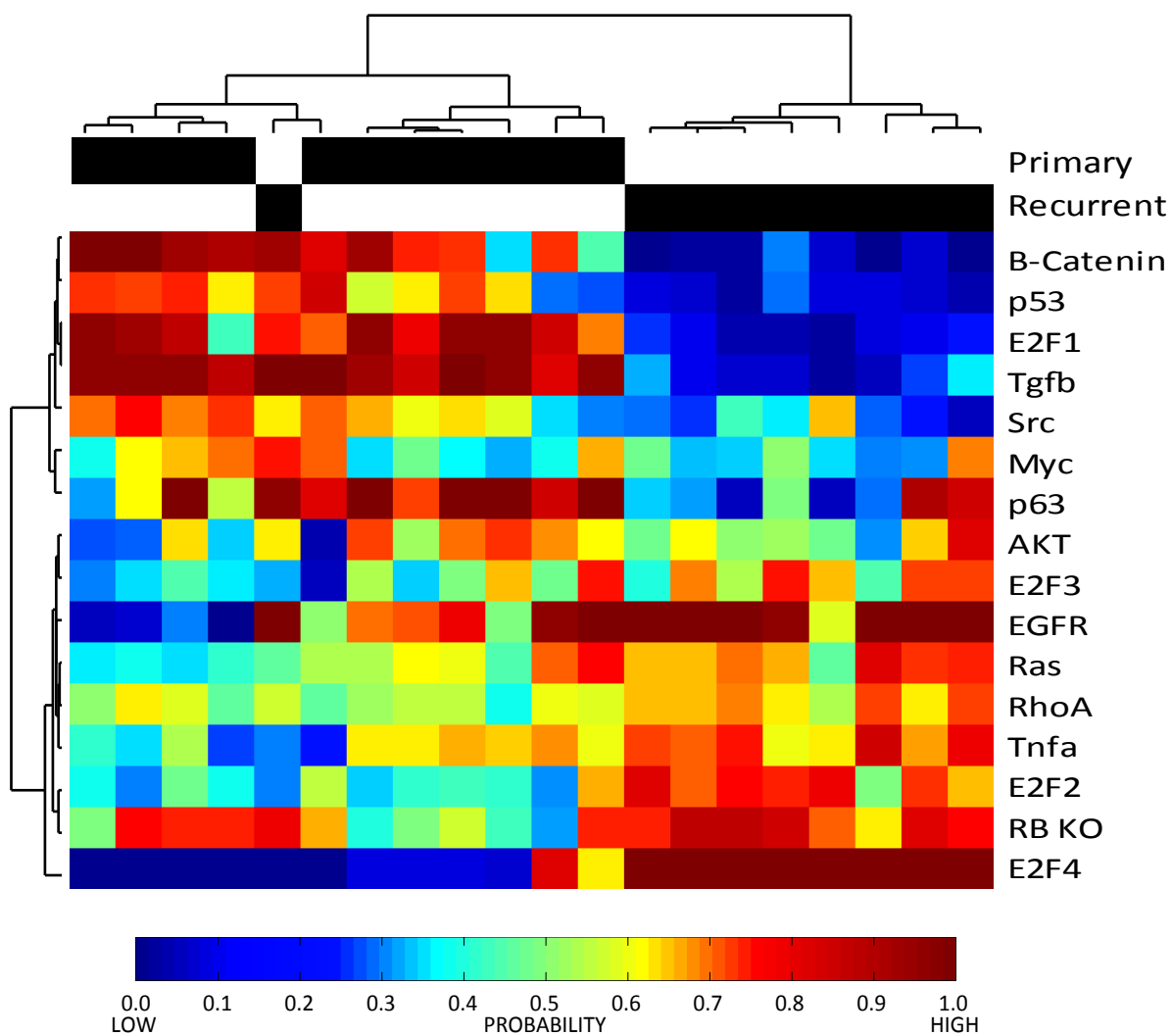

Supplement: Additional file 12 — PDFs of pathway predictions for each mouse model of breast cancer, folders exist for each mouse modelx. [file bcr3672-S12.zip › AdditionalFile12/IGFIR_pathways/HeatmapFigure.pdf]

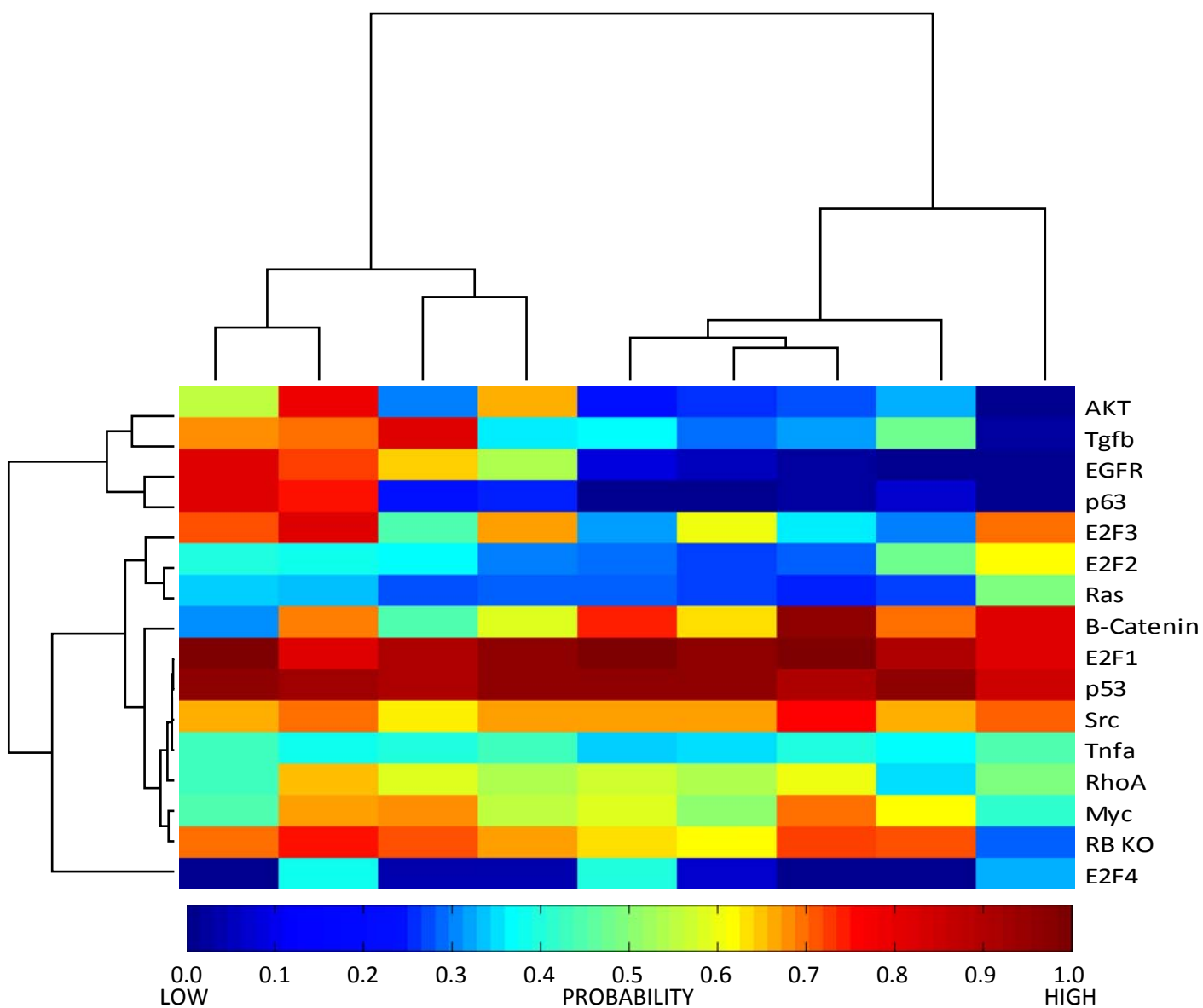

Supplement: Additional file 12 — PDFs of pathway predictions for each mouse model of breast cancer, folders exist for each mouse modelx. [file bcr3672-S12.zip › AdditionalFile12/Int3_pathways/heatmapINT3.pdf]

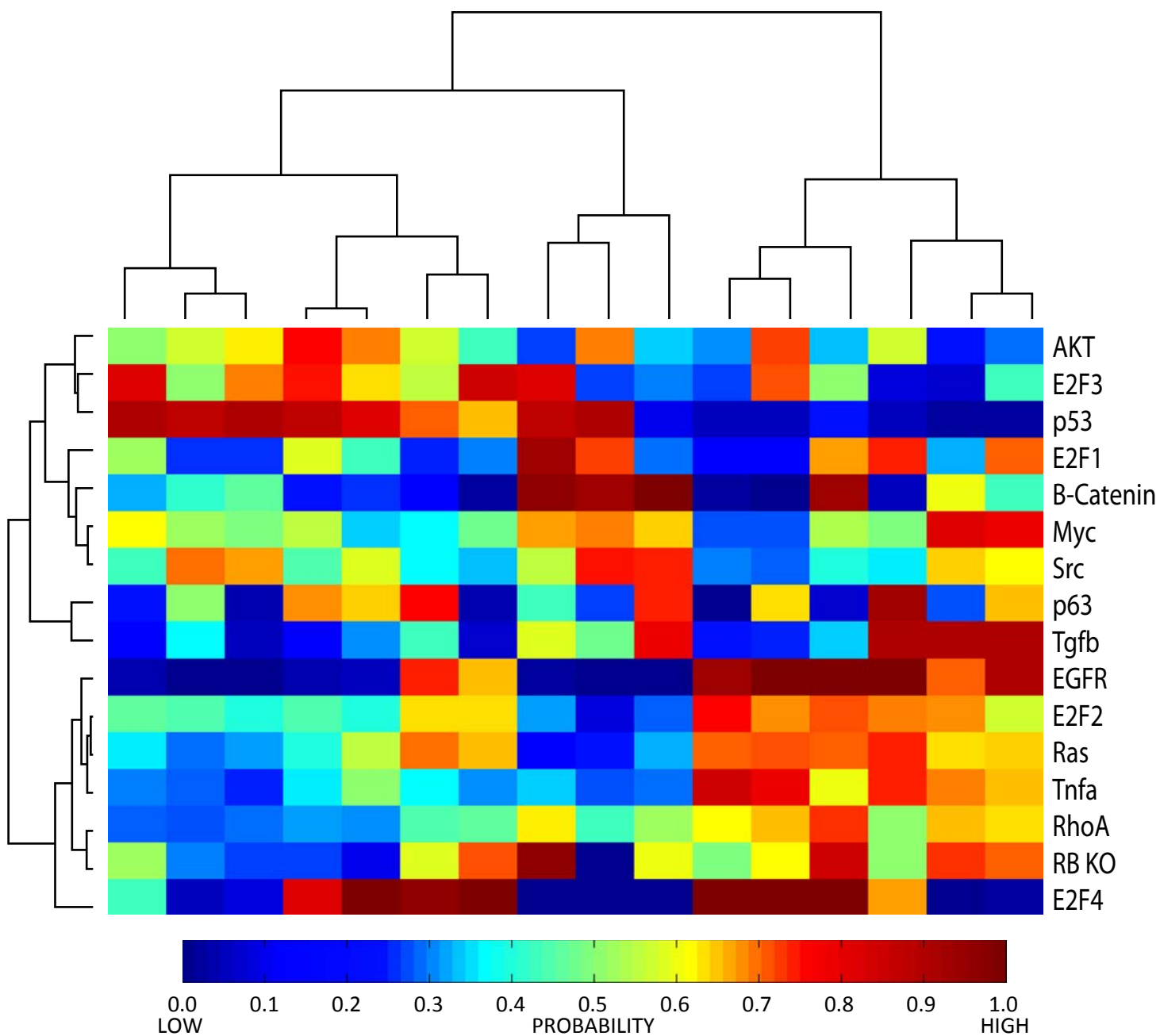

Supplement: Additional file 12 — PDFs of pathway predictions for each mouse model of breast cancer, folders exist for each mouse modelx. [file bcr3672-S12.zip › AdditionalFile12/LPA_pathways/LPA_Heatmap.pdf]

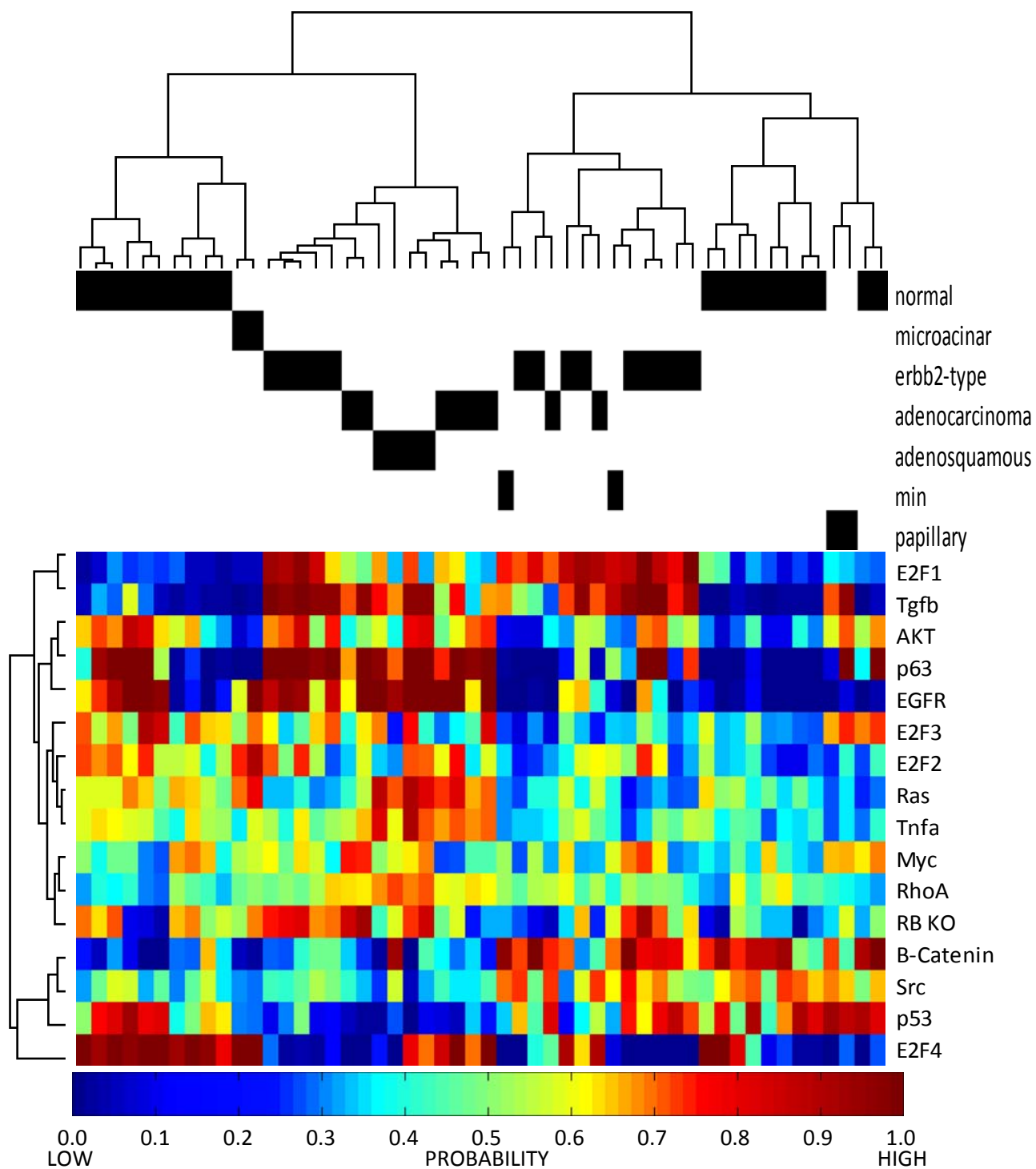

Supplement: Additional file 12 — PDFs of pathway predictions for each mouse model of breast cancer, folders exist for each mouse modelx. [file bcr3672-S12.zip › AdditionalFile12/Met_pathways/heatmap.pdf]

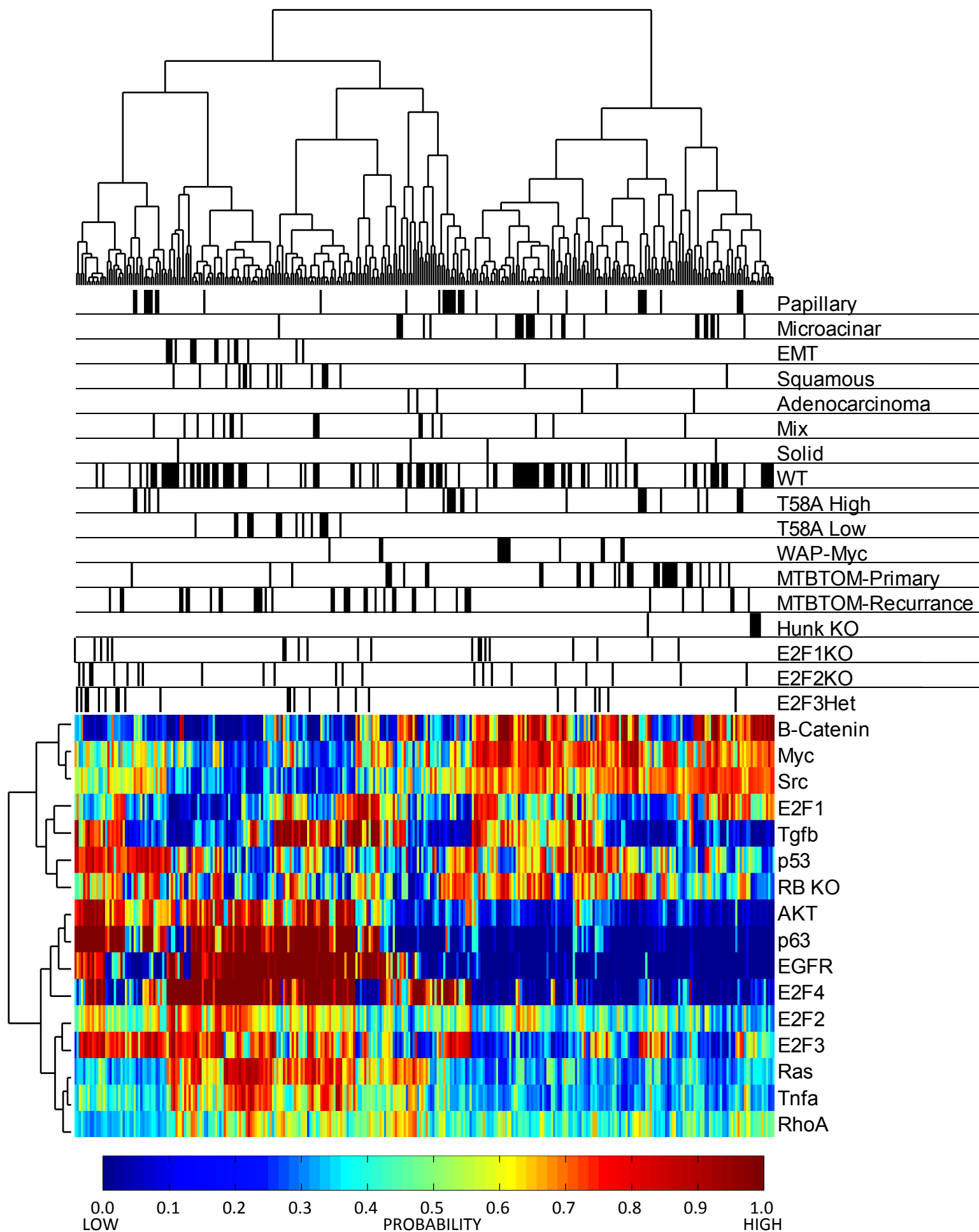

Supplement: Additional file 12 — PDFs of pathway predictions for each mouse model of breast cancer, folders exist for each mouse modelx. [file bcr3672-S12.zip › AdditionalFile12/Myc_pathways/heatmap.pdf]

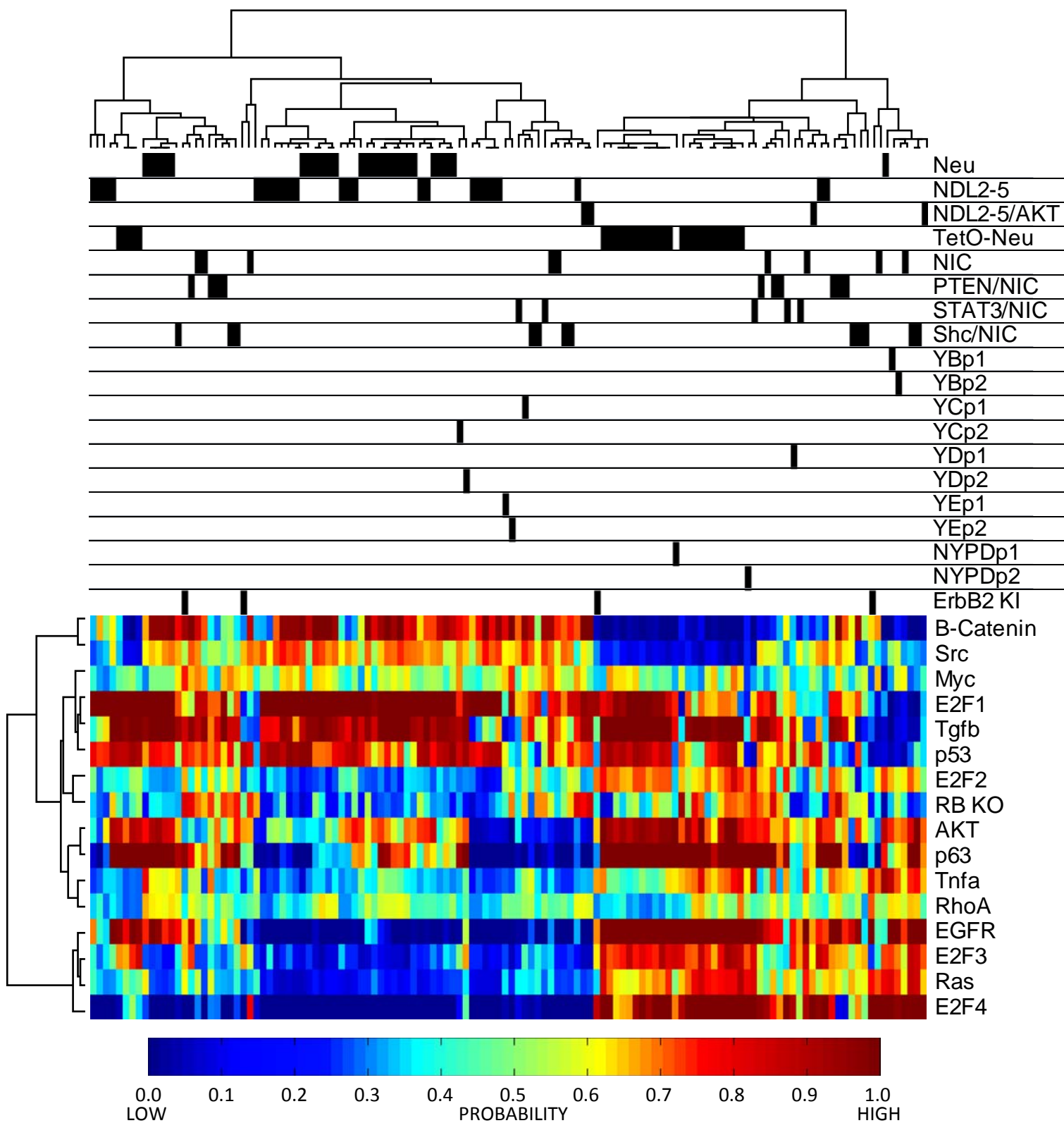

Supplement: Additional file 12 — PDFs of pathway predictions for each mouse model of breast cancer, folders exist for each mouse modelx. [file bcr3672-S12.zip › AdditionalFile12/Neu_pathways/Heatmap.pdf]

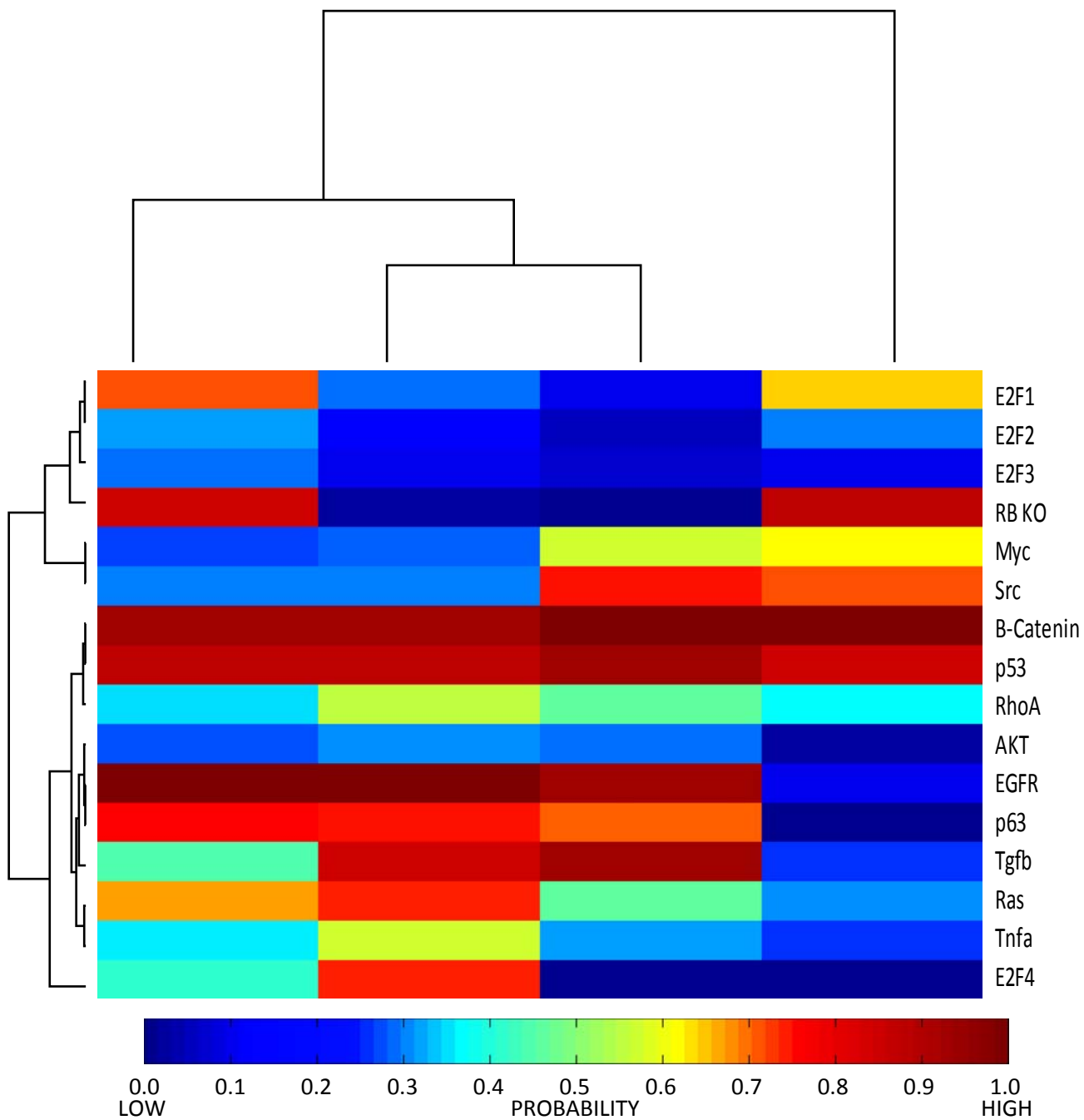

Supplement: Additional file 12 — PDFs of pathway predictions for each mouse model of breast cancer, folders exist for each mouse modelx. [file bcr3672-S12.zip › AdditionalFile12/Notch_Pathways/Pathways_NOTCH.pdf]

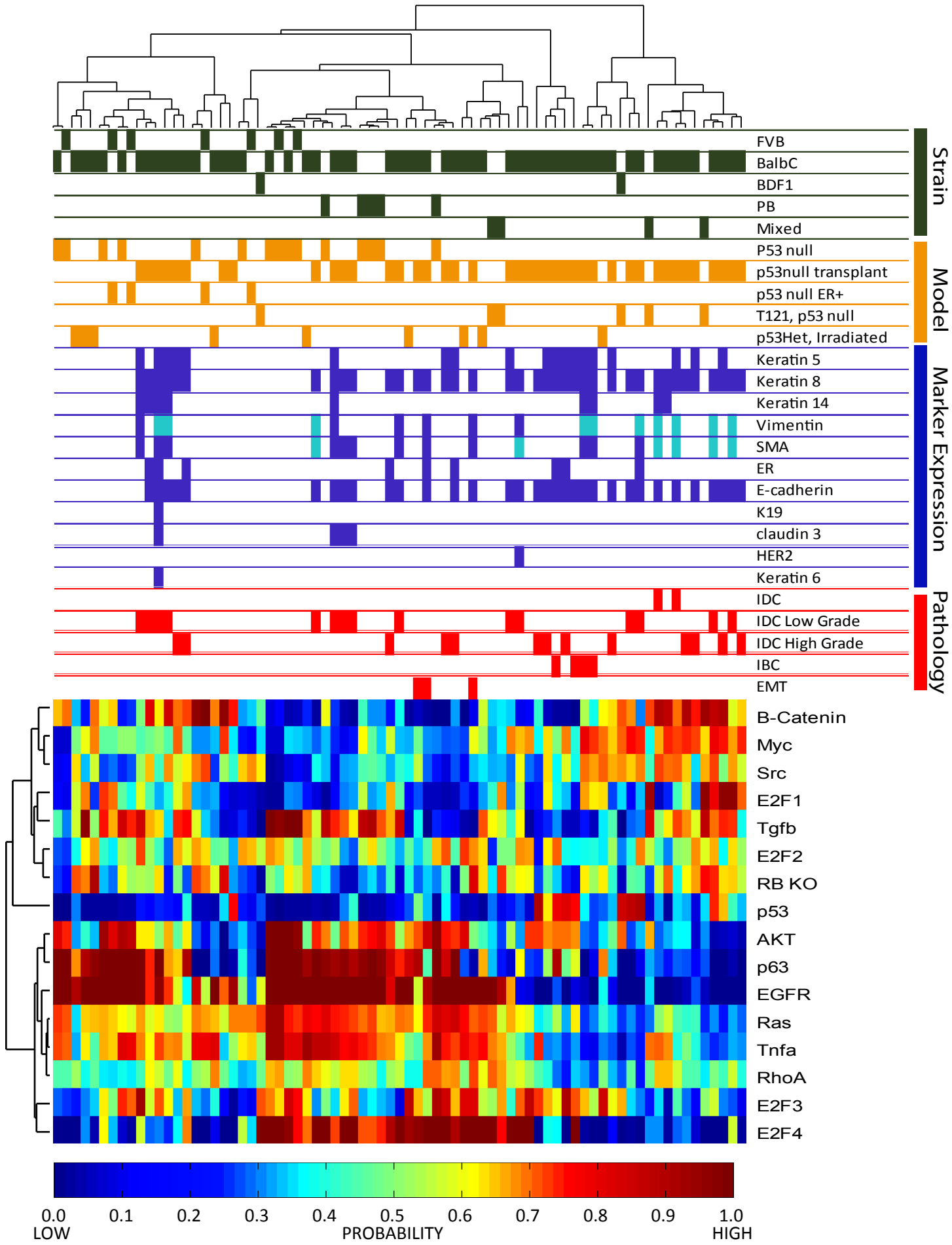

Supplement: Additional file 12 — PDFs of pathway predictions for each mouse model of breast cancer, folders exist for each mouse modelx. [file bcr3672-S12.zip › AdditionalFile12/p53Mutant_pathways/p53_mutantPathways.pdf]

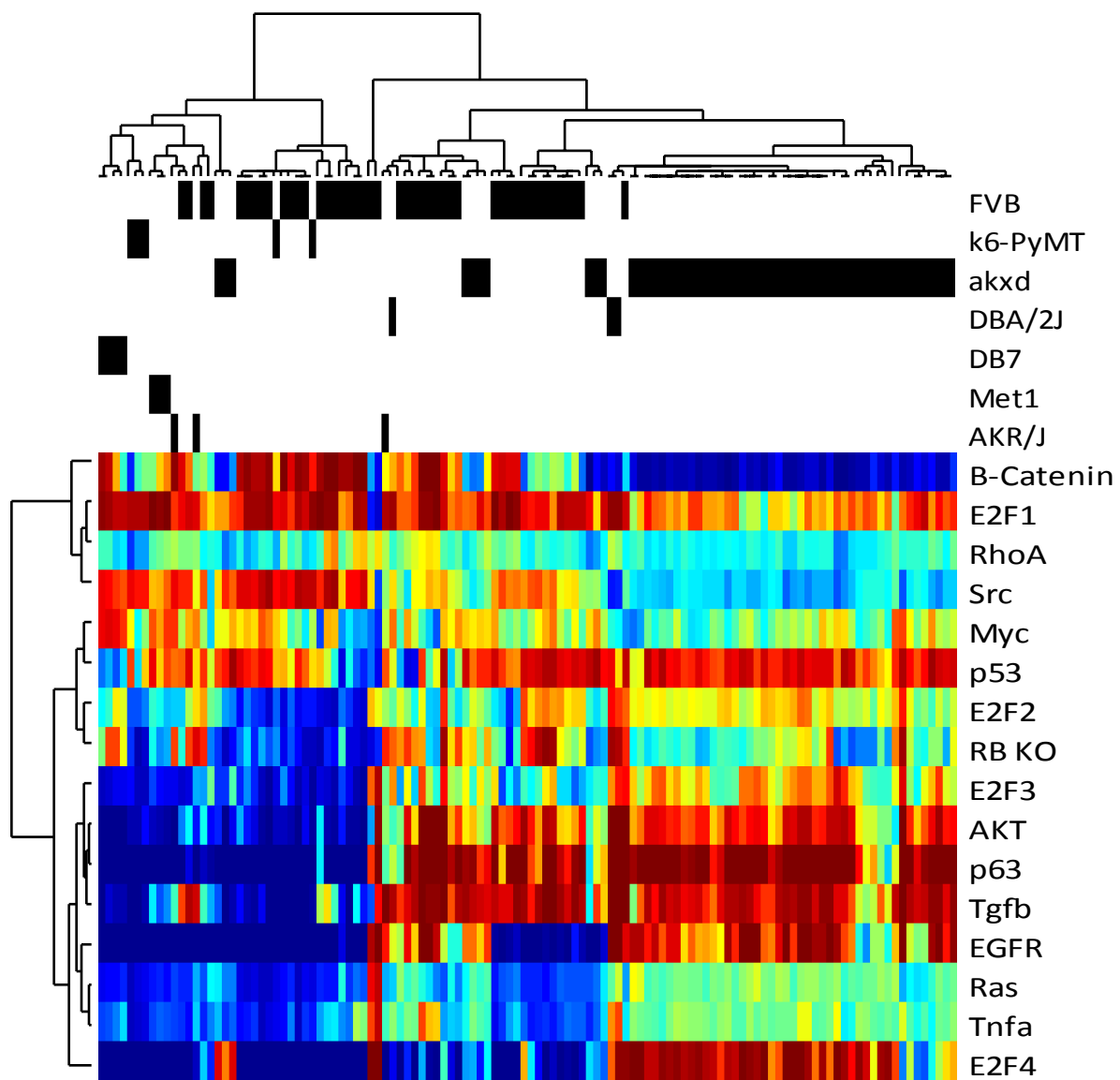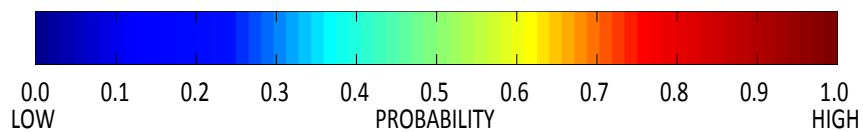

Supplement: Additional file 12 — PDFs of pathway predictions for each mouse model of breast cancer, folders exist for each mouse modelx. [file bcr3672-S12.zip › AdditionalFile12/PyMT_pathways/heatmap.pdf]

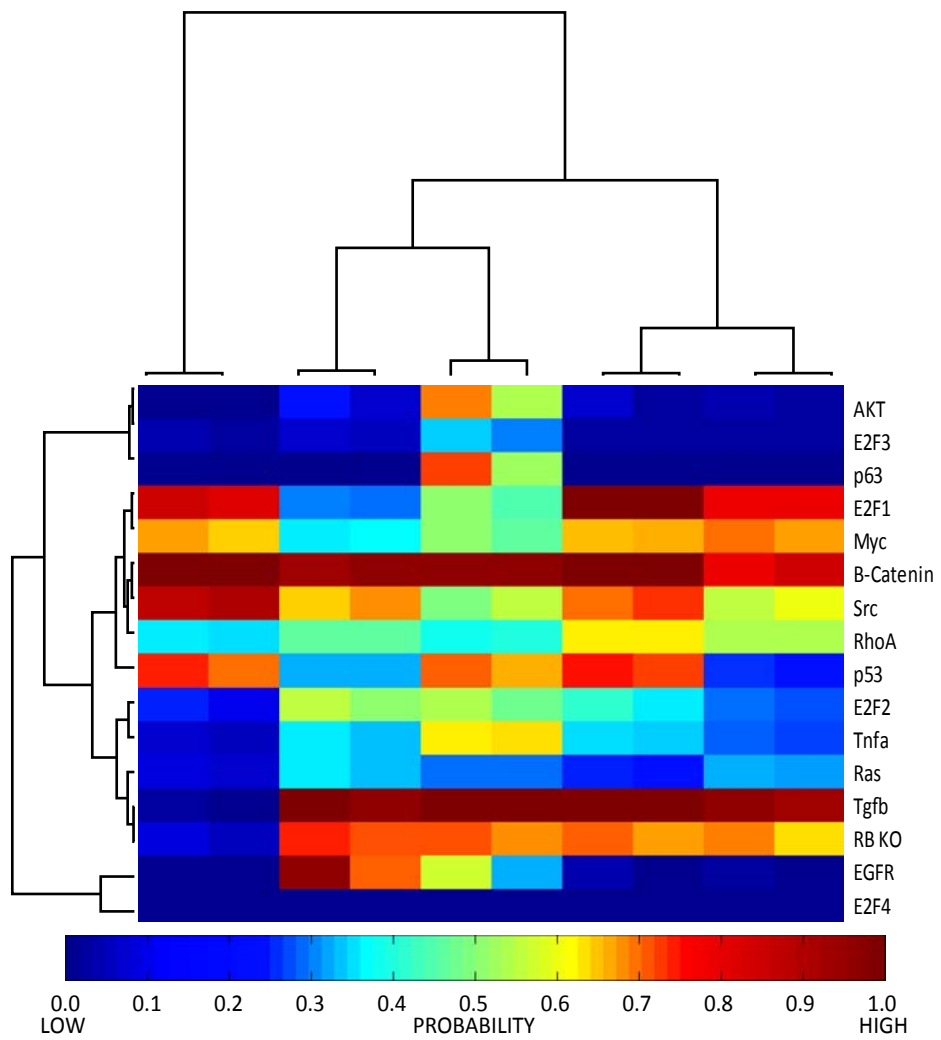

Supplement: Additional file 12 — PDFs of pathway predictions for each mouse model of breast cancer, folders exist for each mouse modelx. [file bcr3672-S12.zip › AdditionalFile12/Ras_pathways/heatmapRas.pdf]

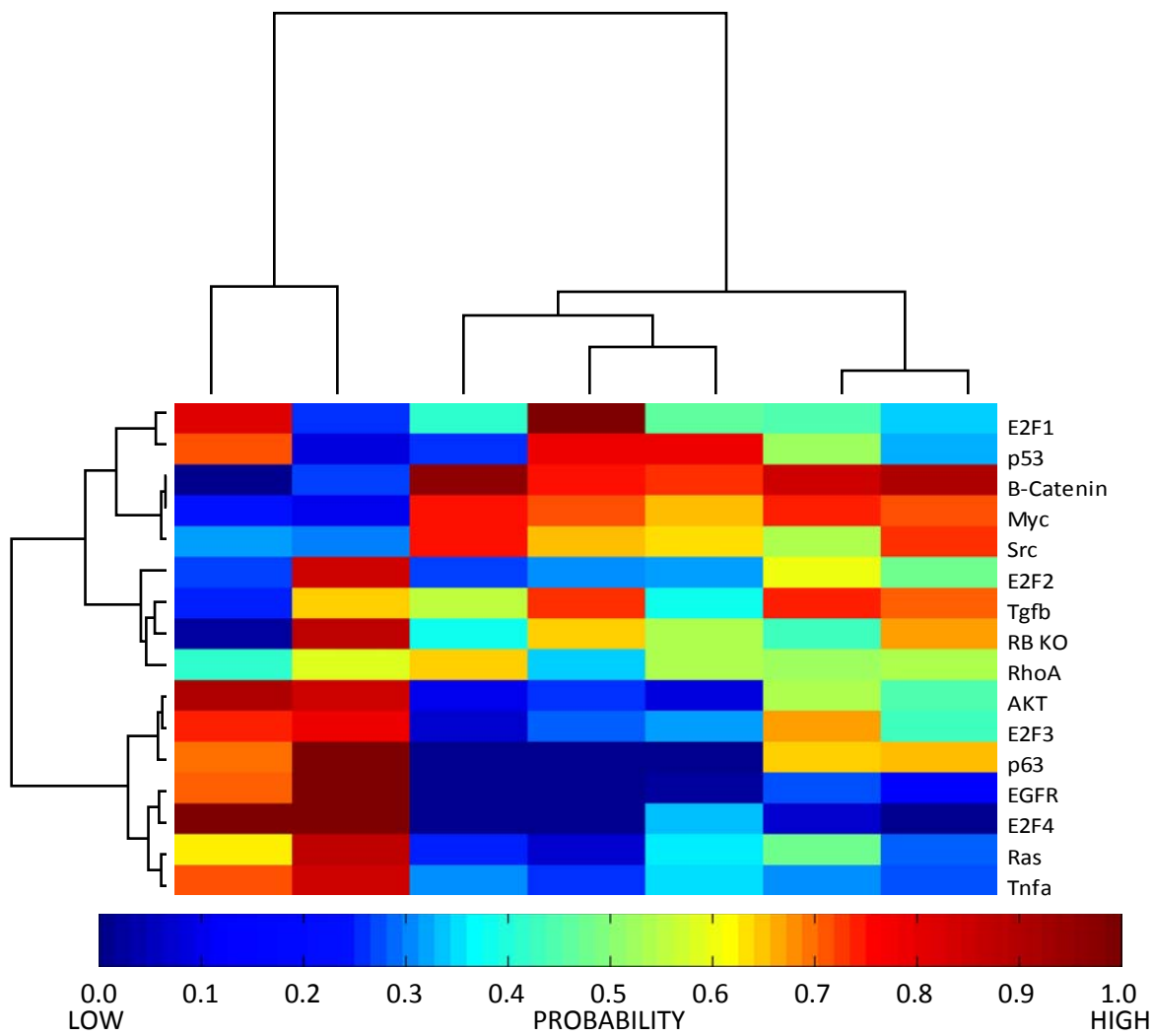

Supplement: Additional file 12 — PDFs of pathway predictions for each mouse model of breast cancer, folders exist for each mouse modelx. [file bcr3672-S12.zip › AdditionalFile12/RBKO_p107mutant_PATHWAYS/heatmap.pdf]

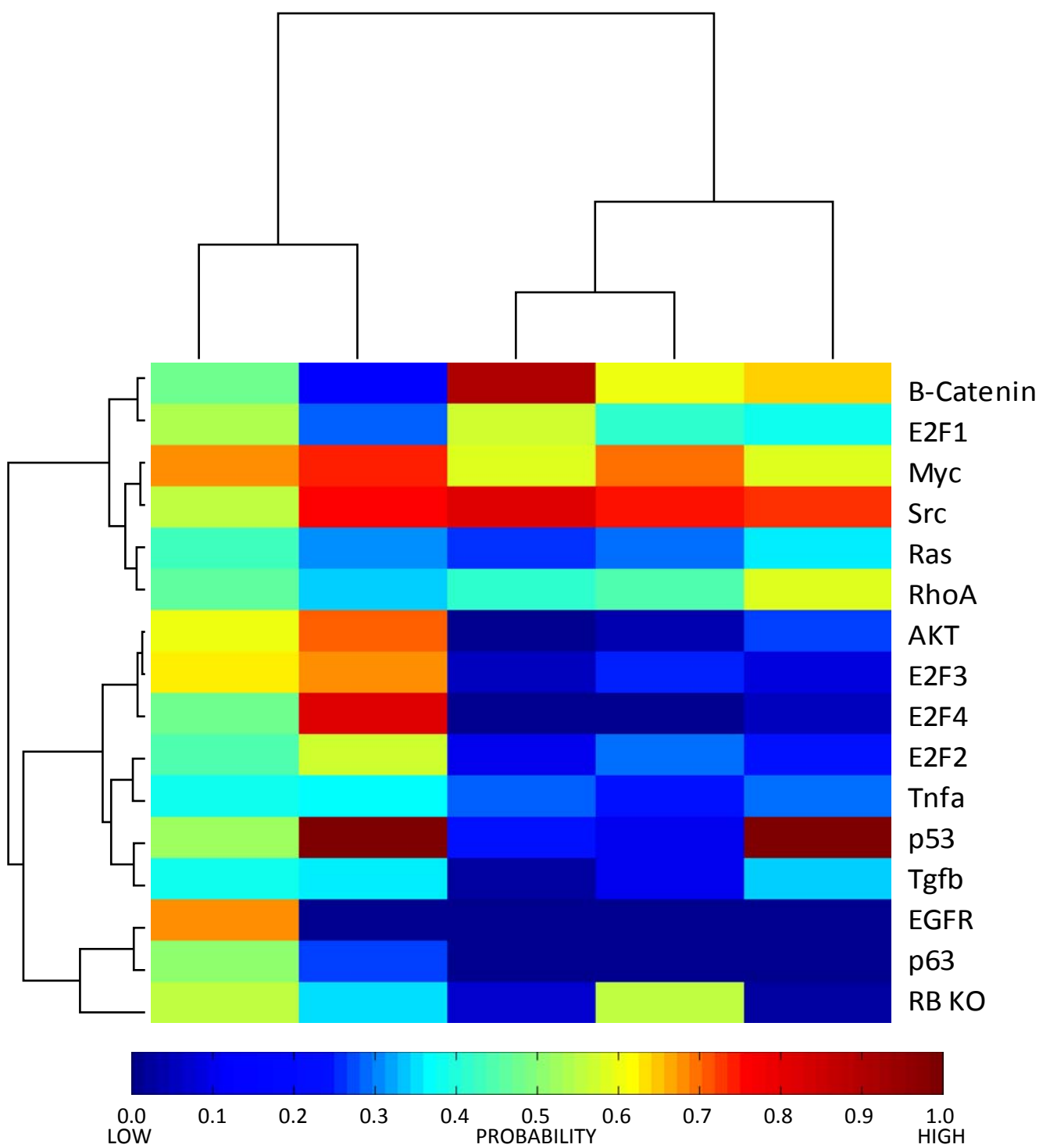

Supplement: Additional file 12 — PDFs of pathway predictions for each mouse model of breast cancer, folders exist for each mouse modelx. [file bcr3672-S12.zip › AdditionalFile12/Stat1KO_Pathways/heatmap.pdf]

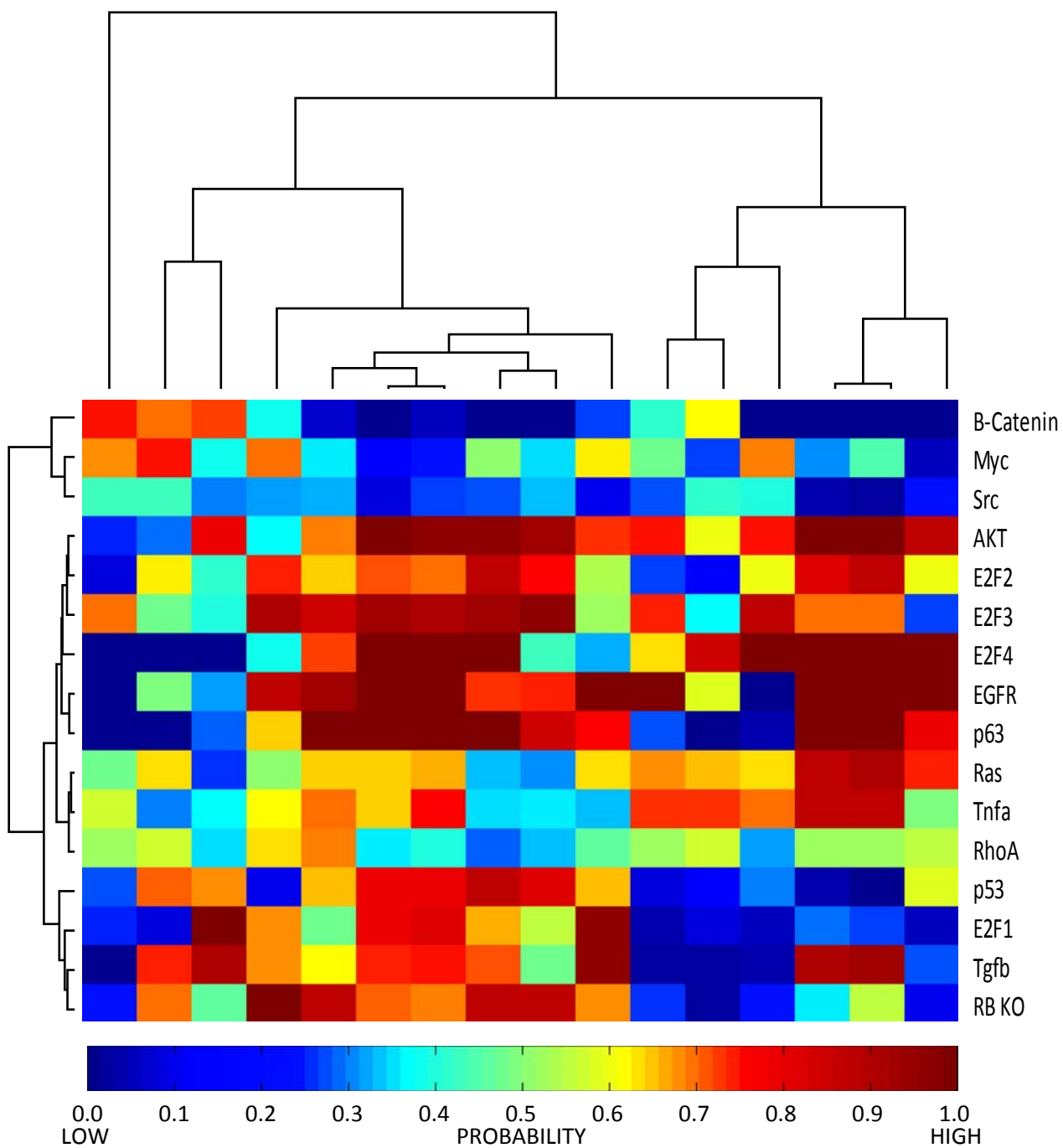

Supplement: Additional file 12 — PDFs of pathway predictions for each mouse model of breast cancer, folders exist for each mouse modelx. [file bcr3672-S12.zip › AdditionalFile12/Stat5_Pathways/heatmap.pdf]

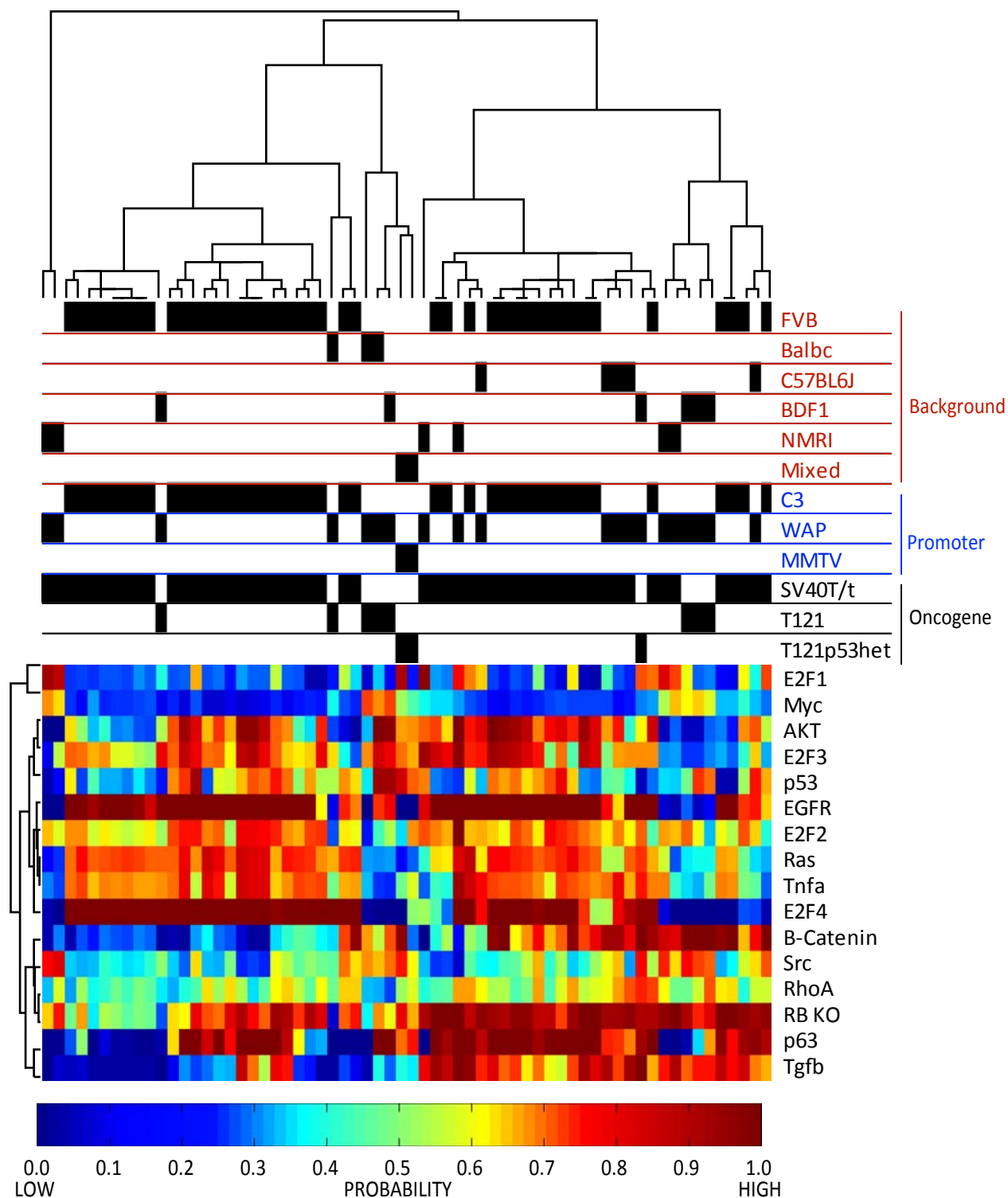

Supplement: Additional file 12 — PDFs of pathway predictions for each mouse model of breast cancer, folders exist for each mouse modelx. [file bcr3672-S12.zip › AdditionalFile12/TAG_pathways/HeatmapPathwayTAG.pdf]

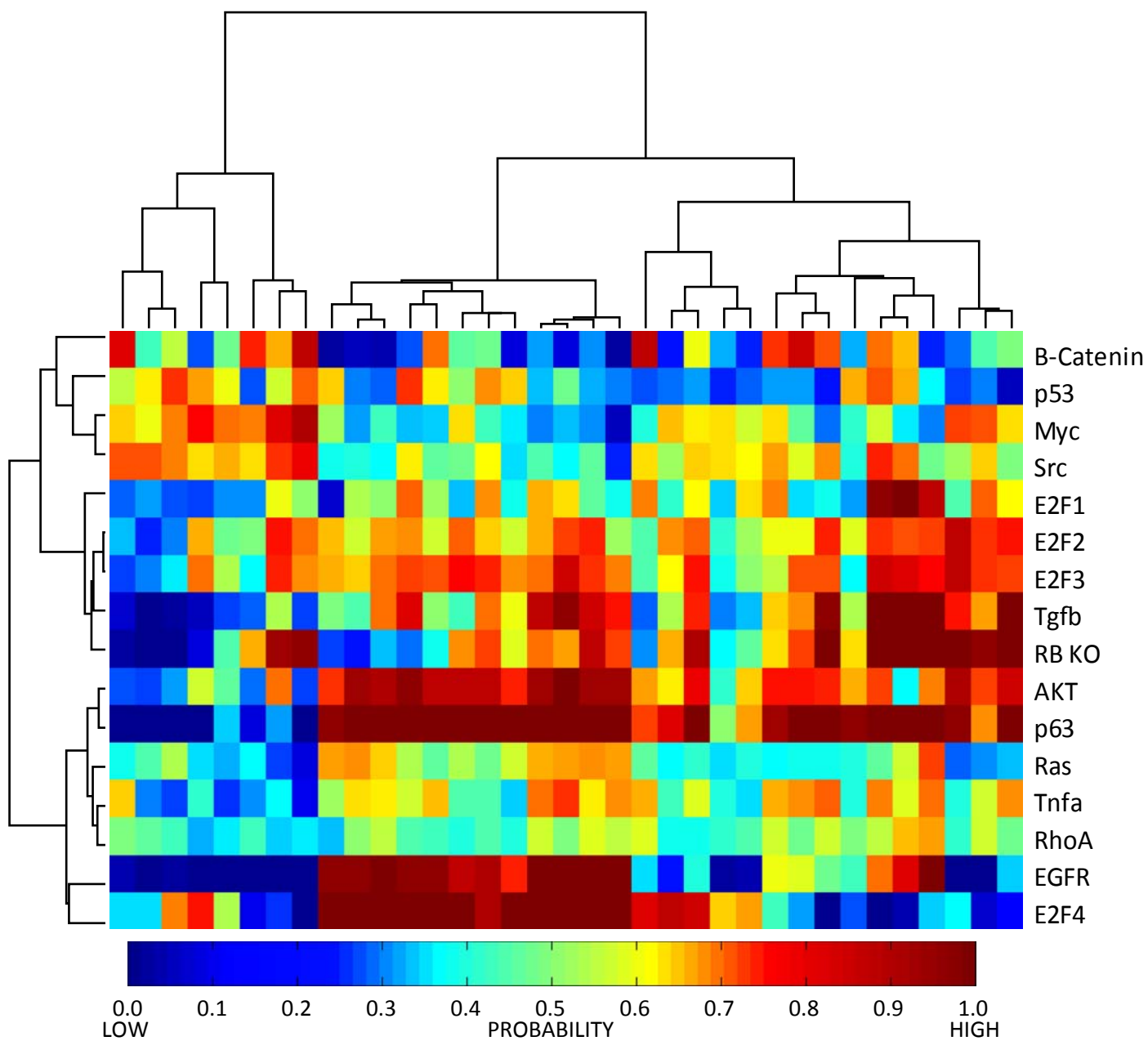

Supplement: Additional file 12 — PDFs of pathway predictions for each mouse model of breast cancer, folders exist for each mouse modelx. [file bcr3672-S12.zip › AdditionalFile12/TNP8_pathways/heatmap.pdf]

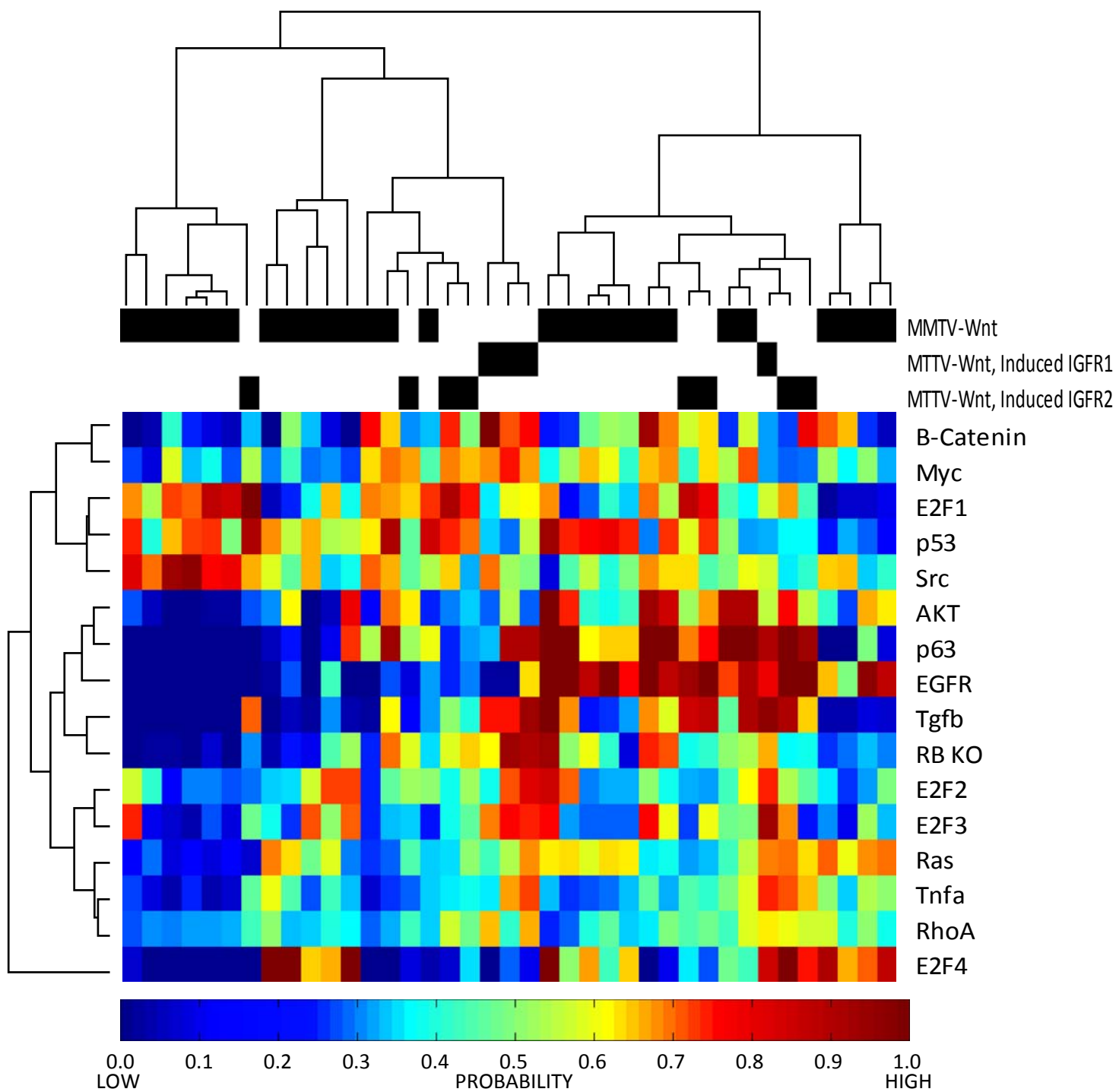

Supplement: Additional file 12 — PDFs of pathway predictions for each mouse model of breast cancer, folders exist for each mouse modelx. [file bcr3672-S12.zip › AdditionalFile12/wnt_pathways/heatmap.pdf]

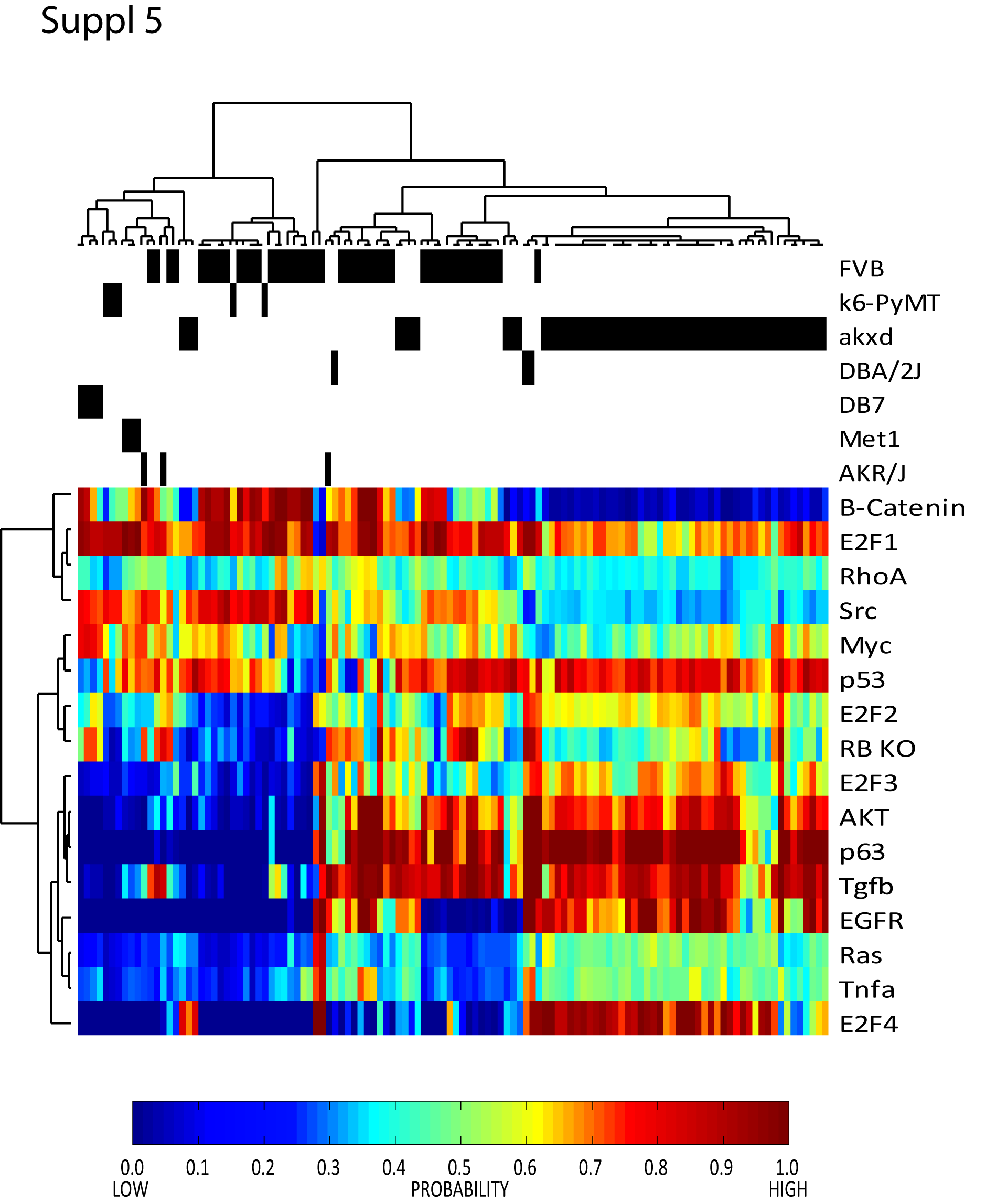

Supplement: Additional file 13: Figure S5 — Unsupervised hierarchical clustering of pathway probabilities for PyMT induced tumors. The dendrogram across the top illustrates the relationship between PyMT tumor types on the basis of pathway activation profiles. Below the dendrogram black bars correspond to sample details on the same line, annotating the genetic background and sample type for each sample. The heatmap shows the predicted pathway activity according to the probabilities listed on the color bar below the heatmap. Directly beside the heatmap, a vertical dendrogram illustrates the degree of correlation between pathways across the samples. [file bcr3672-S13.tiff]

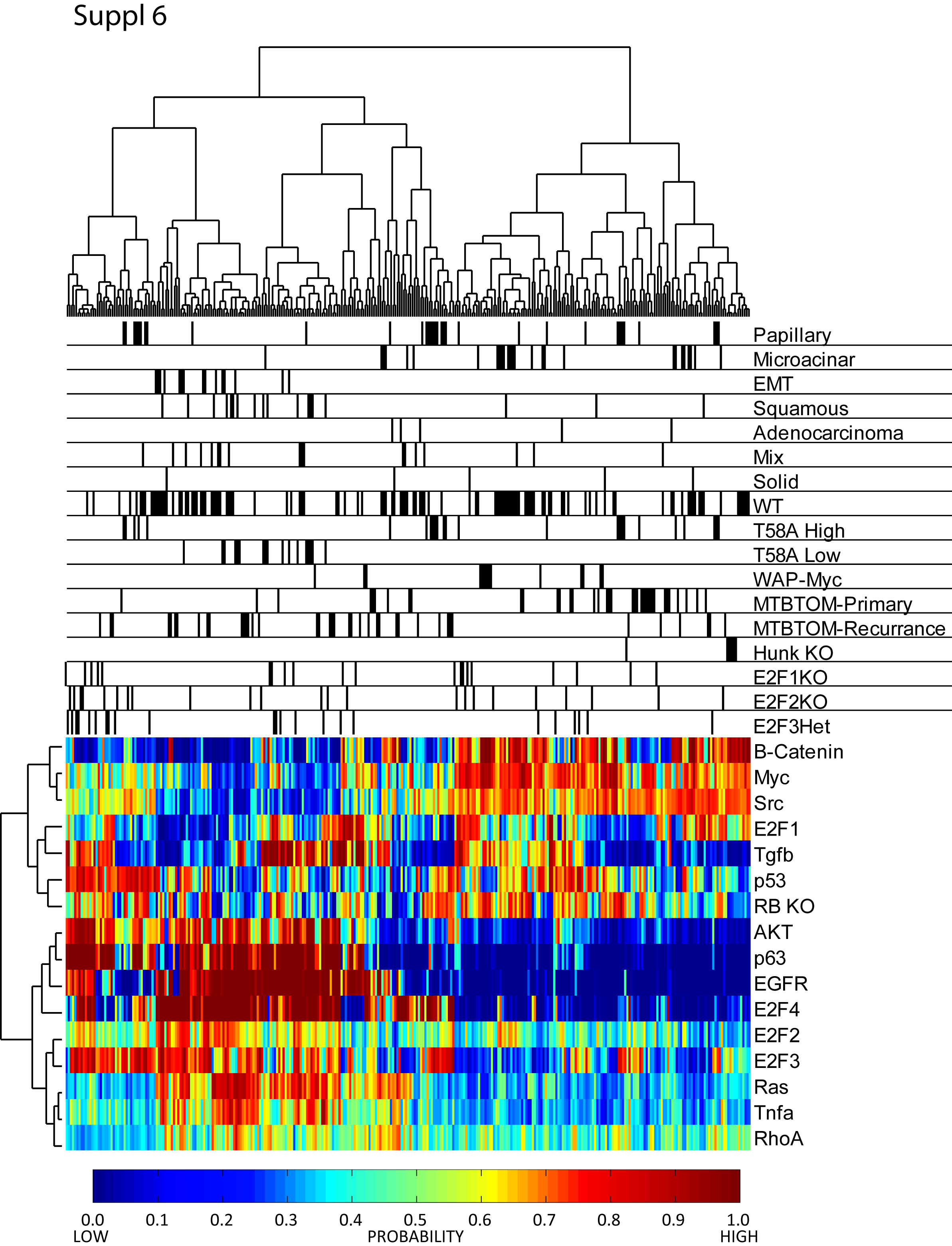

Supplement: Additional file 14: Figure S6 — Unsupervised hierarchical clustering of pathway probabilities for Myc induced tumors. The dendrogram across the top illustrates the relationship between Myc tumor types on the basis of pathway activation profiles. Below the dendrogram black bars correspond to sample details on the same line, annotating the tumor histology (if known), specific form of Myc expression, recurrence status, and additional modifications. The heatmap shows the predicted pathway activity according to the probabilities listed on the color bar below the heatmap. Directly beside the heatmap, a vertical dendrogram illustrates the degree of correlation between pathways across the samples. [file bcr3672-S14.tiff]

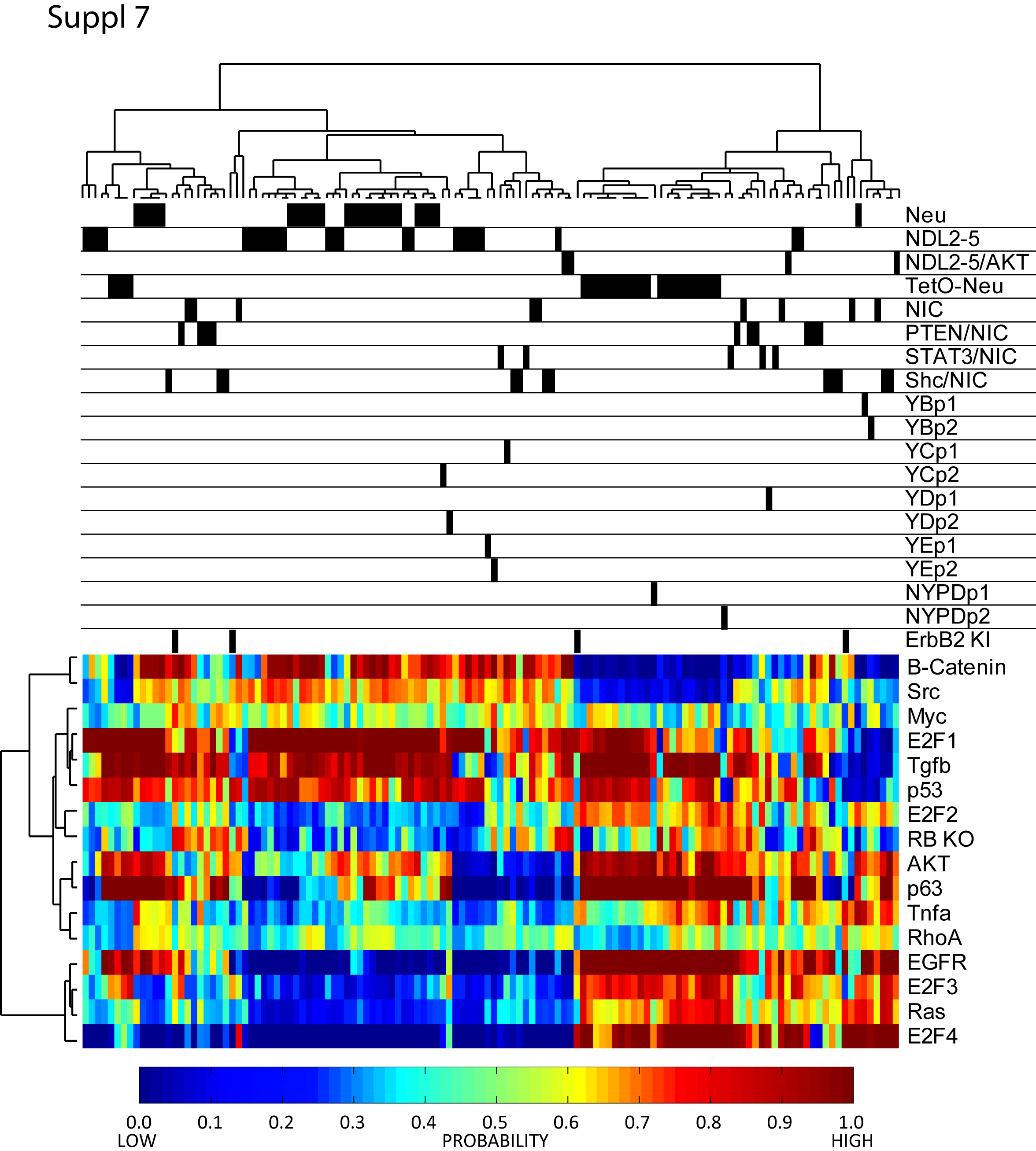

Supplement: Additional file 15: Figure S7 — Unsupervised hierarchical clustering of pathway probabilities for Neu induced tumors. The dendrogram across the top illustrates the relationship between Neu tumor types on the basis of pathway activation profiles. Below the dendrogram black bars correspond to sample details on the same line, annotating the specific form of Neu, and additional modifications. The heatmap shows the predicted pathway activity according to the probabilities listed on the color bar below the heatmap. Directly beside the heatmap, a vertical dendrogram illustrates the degree of correlation between pathways across the samples. [file bcr3672-S15.tiff]

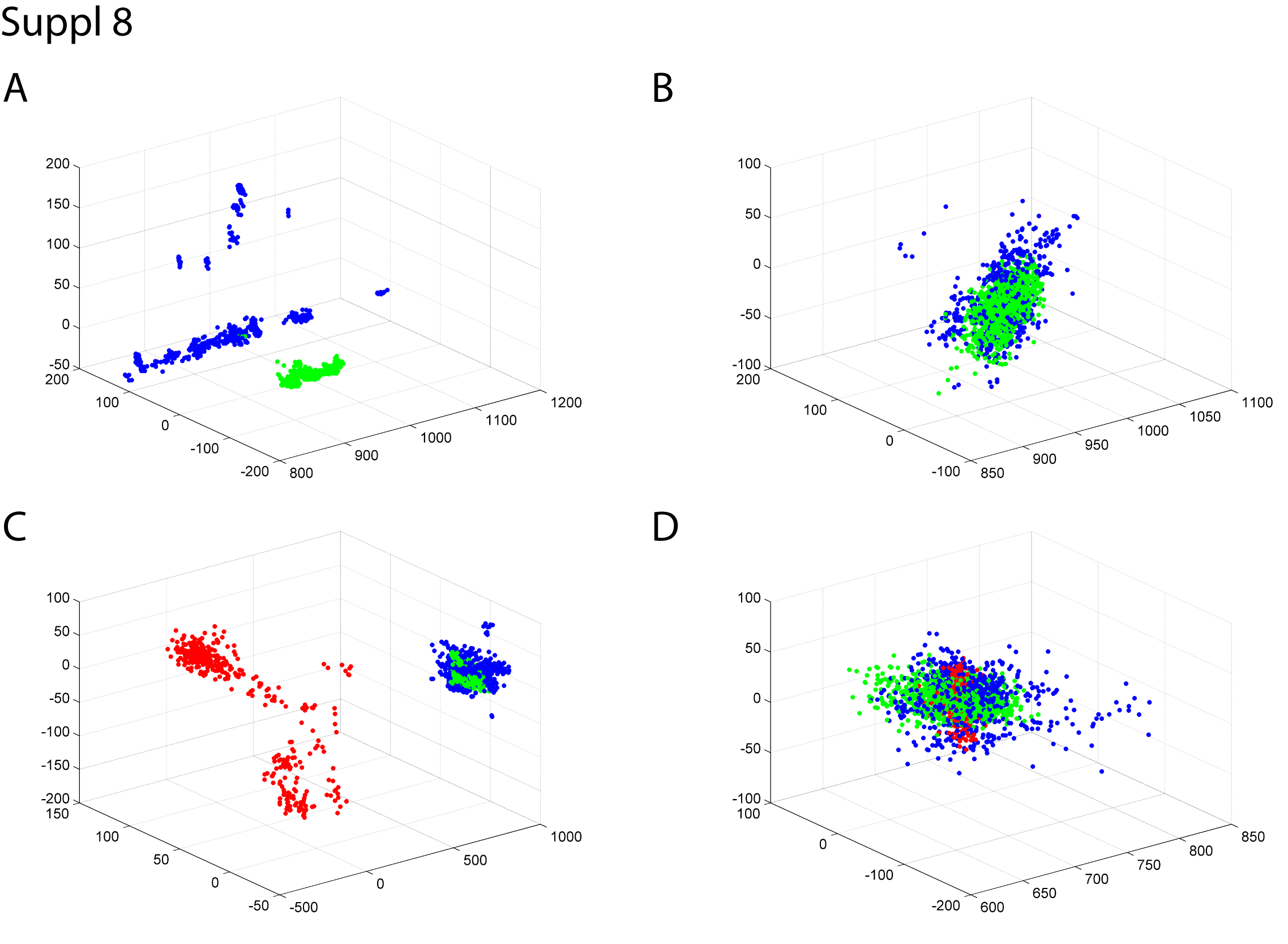

Supplement: Additional file 16: Figure S8 — Removal of batch effects between mouse and human breast cancer datasets. (A) Mouse (blue) and human (green) Affymetrix data gene expression variance plotted onto three principle components prior to BFRM. (B) Mouse (blue) and human (green) Affymetrix data gene expression variance plotted onto three principle components after BFRM. (C) Human(green) and mouse (blue) Affymetrix data after BFRM correction put together with mouse Agilent data (red) prior to COMBAT. (D) Human(green) and mouse (blue) Affymetrix data after BFRM correction put together with mouse Agilent data (red) after COMBAT artifact correction. [file bcr3672-S16.tiff]

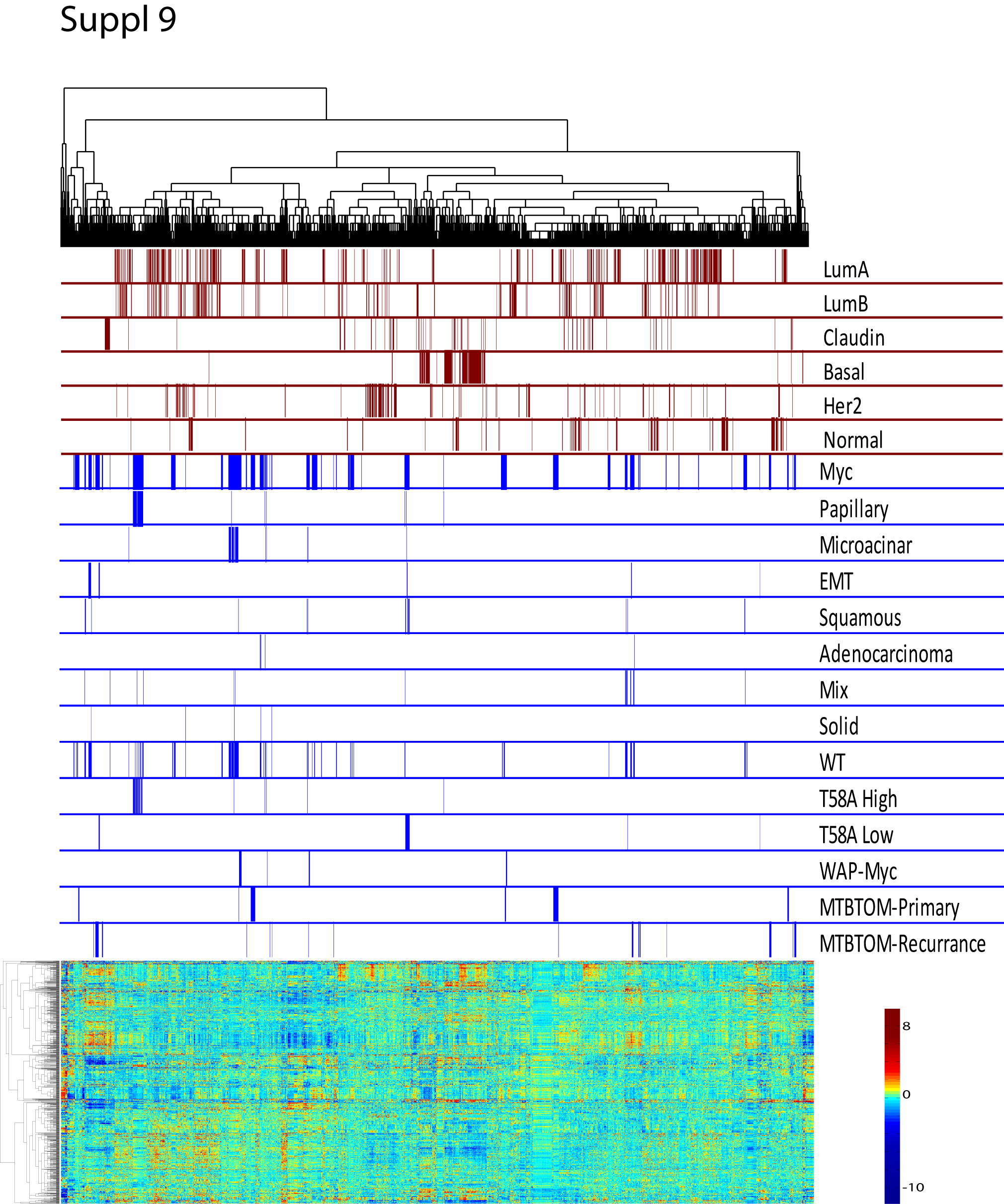

Supplement: Additional file 17: Figure S9 — Unsupervised hierarchical clustering of Myc mouse mammary tumors and human breast cancer gene expression data. Across the top, the dendrogram illustrates the relationship between human and mouse tumor samples on the basis of gene expression profiles. The red bars mark the intrinsic subtype of each human tumor sample according to the annotation on the same line. The blue bars correspond to the Myc mouse mammary tumor type. Below this, a heatmap shows the gene expression patterns for each sample, with expression values illustrated according to the color bar on the right. The dendrogram beside the heatmap shows the correlation between genes based on expression patterns across the samples in the dataset. [file bcr3672-S17.tiff]

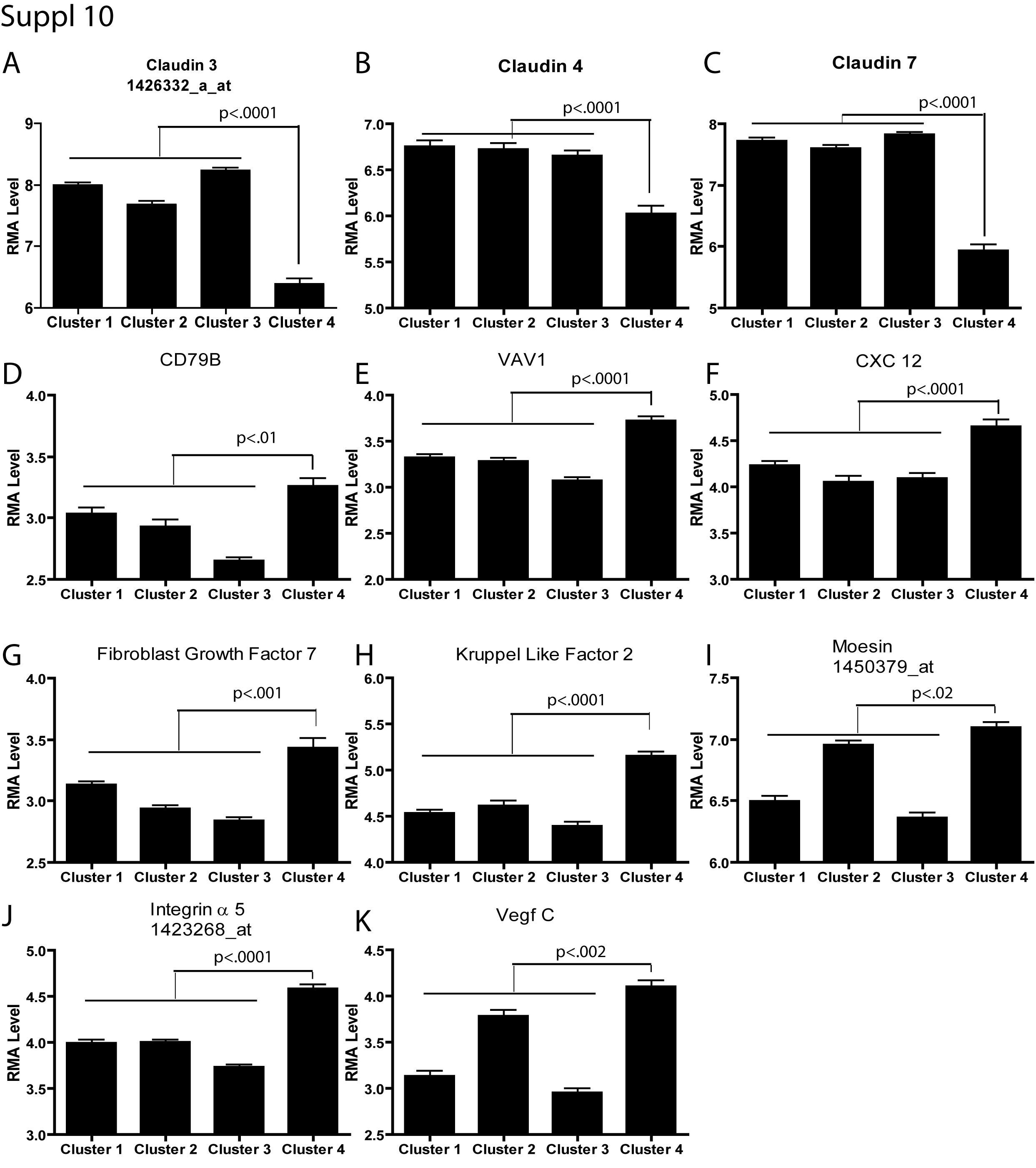

Supplement: Additional file 18: Figure S10 — Claudin low marker expression in the black cluster mouse mammary tumors. Claudin low marker expression comparisons for cluster 4 (black) tumors compared to tumors in all other clusters as defined by Figure 1A. (A-C) Cell adhesion markers that have low expression in claudin low human tumors are also down regulated in cluster 4 (black tumors), P <.0001. (D-E) Genes that are involved with the immune system that are found to be highly expressed in claudin low human tumors are highly expressed in mouse cluster 4 tumors (black), P <.01 for CD79B and P <.0001 for VAV1. (F) Chemokine (C-X-C motif) ligand 12, involved in cell communication and previously shown to be highly expressed in claudin low tumors, is upregulated in cluster 4 (black) mouse mammary tumors, P <.0001. (G) Fibroblast growth factor 7, an extracellular matrix related factor and previously shown to be highly expressed in claudin low tumors, is upregulated in cluster 4 (black) mouse mammary tumors, P <.0001. (H-J) Cell migration markers previously shown to be highly expressed in human claudin low tumors are upregulated in mouse cluster 4(black) tumors, P <.02 for moesin and P <.0001 for integrin α5. (K) Angiogenesis marker, VEGFC, was previously shown to be upregulated in human claudin low tumors and is highly expressed in mouse cluster 4(black) tumors. [file bcr3672-S18.tiff]

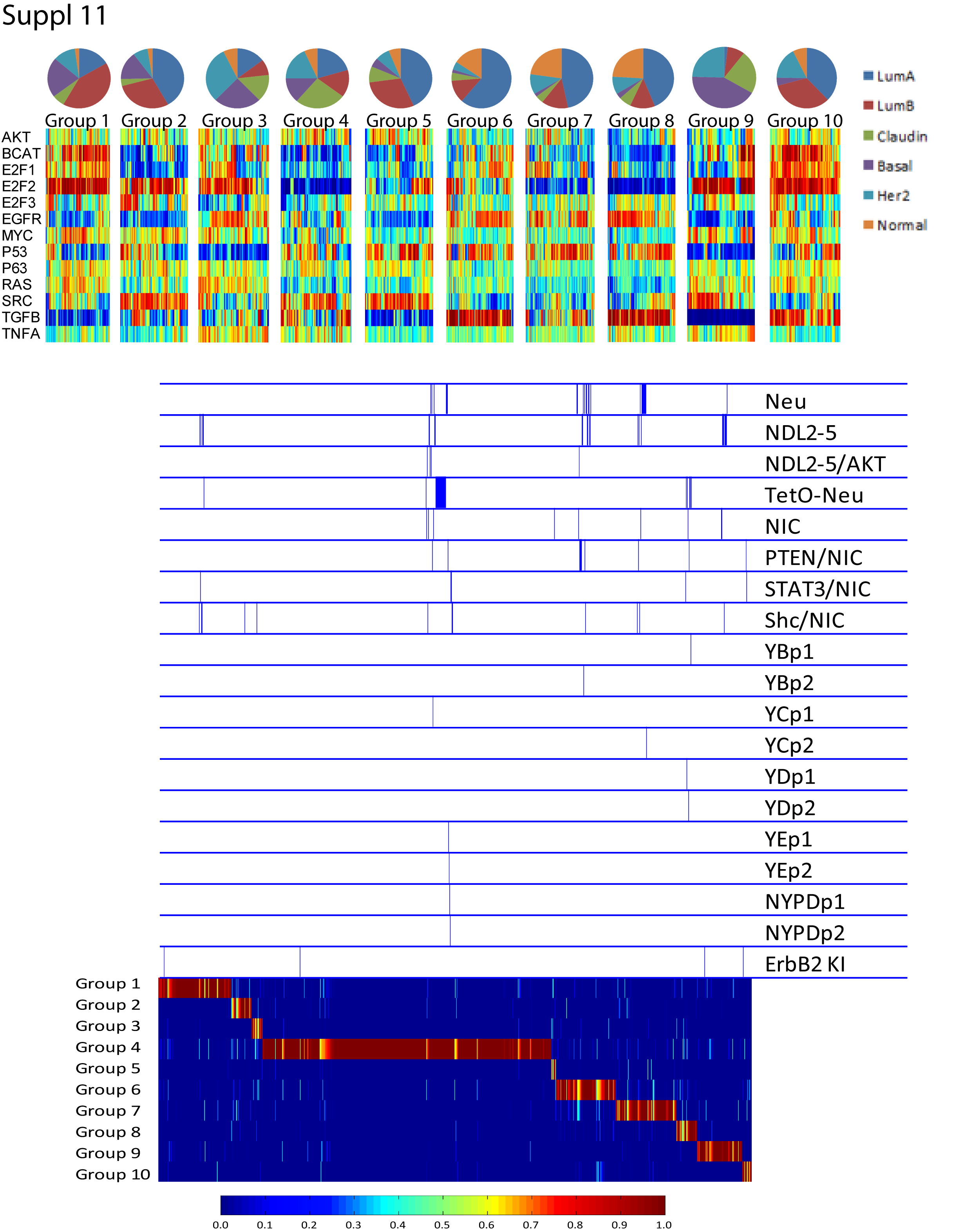

Supplement: Additional file 19: Figure S11 — Mixture modeling highlighting pathway relationships between human breast cancer and specific models of Neu mediated tumorigenesis. Pie charts above each heatmap illustrate the distribution of the intrinsic subtype of samples in each group, according to the color-coded legend. The heatmap for groups 1 to 10 show predicted pathway activity with probabilities corresponding to the color bar at the bottom of the figure. Below this, blue bars mark the samples corresponding to annotations on the same line. Following the samples down to the heatmap below the blue bars, the probability that a specific type of Neu model has similar pathway activation profiles is shown for each group. Probabilities for this heatmap are shown according to the color bar at the bottom of the figure. [file bcr3672-S19.tiff]
