# Supplementary material for: A genomic analysis of mouse models of breast cancer reveals molecular features of mouse models and relationships to human breast cancer
Source: Breast Cancer Res. 2014 Jun 5;16(3):R59. doi: 10.1186/bcr3672 (PMC4078930; doi:10.1186/bcr3672)
Supplement: Additional file 11 — GSEA for mouse models compared to all other models or to mammary gland development. Listed by model. [file bcr3672-S11.zip › AdditionalFile11/Stat5/vs_normal_mammary/Note.docx]

C3: None of the gene sets passed
